# Orange-Derived Extracellular Vesicles: Characterization and Therapeutic Applications in Normal and Diabetic Wound Healing in In Vivo Models

**DOI:** 10.3390/cells15030244

**Published:** 2026-01-27

**Authors:** Chiara Gai, Margherita Alba Carlotta Pomatto, Federica Negro, Lucia Massari, Maria Chiara Deregibus, Massimo Cedrino, Cristina Grange, Alessandro Burello, Joanna Kopecka, Ivan Molineris, Anel Ordabayeva, Alessandro Damin, Federica Antico, Chiara Riganti, Vito Fanelli, Natasa Zarovni, Giovanni Camussi

**Affiliations:** 1 Department of Medical Sciences, University of Turin, 10126 Turin, Italy; 2 Molecular Biotechnology Center—MBC, University of Turin, Via Nizza 52, 10126 Turin, Italy; 3 Department of Anesthesiology and Intensive Care, University of Turin, 10126 Turin, Italy; 4 Department of Oncology, University of Turin, 10126 Turin, Italy; 5 Department of Life Sciences and Systems Biology & Molecular Biotechnology Center—MBC, University of Turin, Via Nizza 52, 10126 Turin, Italy; 6 Department of Chemistry, NIS Centre and INSTM Reference Centre University of Turin, Via G. Quarello 15, 10135 Turin, Italy; 7 Forb—Fondazione per la Ricerca Biomedica, ONLUS—Molecular Biotechnology Center (MBC), 10126 Turin, Italy; 8 RoseBio Srl, Via Mario Bianco 9, 20131 Milano, Italy

**Keywords:** extracellular vesicle, plant-derived extracellular vesicle, wound healing, citrus sinensis, citrus sinensis extracellular vesicles, diabetes, diabetic ulcer

## Abstract

**Highlights:**

**What are the main findings?**

- Extracellular vesicles derived from orange juice (oEVs) accelerated wound healing in both healthy and diabetic mice when formulated with hydrogels.
- In vitro studies suggest that the regenerative properties of oEVs may be related to stimulation of cell migration, capillary-like structure formation, cell proliferation, and strong antioxidant activity against hyperglycemia and pro-inflammatory conditions.

**What are the implications of the main findings?**

- oEVs show potential as an alternative to human-derived EVs for regenerative medicine.
- oEVs could solve human EV limitations in scalability, variability, safety, and cost.

**Abstract:**

Extracellular vesicles (EVs) of human origin show promise for regenerative medicine and wound healing. However, they have limitations regarding scalability, variability, safety, and costs. Plant-derived EVs may represent a valid alternative. This study investigated the regenerative potential of EVs extracted from orange juice (oEVs). oEVs obtained by standard ultracentrifugation were compared with oEVs purified by tangential flow filtration (TFF), a scalable technique suitable for large-scale and regulatory-compliant manufacturing. Comparisons included size, morphology, pH, Zeta potential, protein and RNA content, Raman spectroscopy, and proteomic, metabolomic, and RNA sequencing. The regenerative potential of oEVs was tested in vitro, with cell migration, endothelial tube formation, and proliferation assays performed. Antioxidant ability was tested on endothelial cells stressed by hyperglycemia or pro-inflammatory cytokine cocktails. Next, oEVs were formulated with different hydrogels and tested at different doses on skin ulcers on healthy and diabetic mice. TFF oEVs showed the same physio-chemical characteristics and a comparable molecular content as those isolated by ultracentrifugation, confirming the path to scalability. In vitro oEVs promoted cell migration, formation of capillary-like structures, cell proliferation, and strong antioxidant activity. Moreover, oEVs effectively accelerated in vivo wound closure in healthy and diabetic mice. Thus, oEVs may provide a useful and cost-effective ingredient for improved and effective wound treatment strategies.

## 1. Introduction

Cell-to-cell communication through the mechanism of vesiculation, which includes the active secretion and uptake of nano- and micro-sized membrane vesicles, is conserved across all domains of life [1,2,3]. These biological particles, collectively named extracellular vesicles (EVs), have been extensively studied for their intrinsic properties and the roles they play in a plethora of physiological and pathological processes. The growing EV research community, gathered within and around the International Society of Extra-cellular Vesicles, starting from 2013 [4], has progressively established standards for the classification and nomenclature of EVs, and minimum requirements for their study and reporting, and has updated and refined these within international MISEV guidelines [5,6] for the accommodation of outstanding EV heterogeneity and complexity. Although EV pleiotropic biological activities have to-date mostly been studied in human and animal sources, EV production from other sources such as procaryotes and plants was observed and reported as early as the 1960s [7,8,9]. Recent studies have elucidated some appealing features of these EV sources that position them as a plausible alternative to mammalian cell-produced EVs for high value applications such as regenerative medicine and other therapeutic purposes.

EVs carry out their biological functions by transporting an assortment of bioactive molecules to recipient cells. Secreted vesicles typically comprise a bilayer lipid membrane derived from the originating cell, and diverse molecular cargo, comprising bio-active lipids, proteins, and nucleic acids [1,2,3,4,5,6]. This cargo endows the EVs with the ability to modulate critical pathways and exert therapeutic capability via intercellular signaling [9,10]. It has been demonstrated that EVs derived from stem cells can accelerate the natural process of cutaneous wound healing [11,12,13]. Cutaneous wound repair involves a complex, dynamic series of physiological events that collectively contribute to the well-orchestrated recovery of injured skin tissue [14,15]. Over the last decade, and especially in the recent couple of years, an overwhelming number of studies have reported evidence of anti-inflammatory, anti-aging, and wound healing effects of mesenchymal stem cell (MSC)-derived EVs in in vivo and in vitro models, suggesting multiple mechanisms by which these human cell secretome fractions can facilitate each stage of skin wound healing [16,17,18,19,20]. These refer to their potential to modulate many of the migration, inflammatory, and proliferative functions of different cell types that are involved in and necessary for skin repair, including fibroblasts, immune cells, endothelial cells, and keratinocytes. Additionally, mesenchymal stem cell-derived EVs may help to minimize scarring during the wound healing process [18]. Therefore, MSC-derived EV factors are now widely accepted and studied as a new generation of cell-free therapeutics, with emerging research and commercial work focusing on advanced dermatology and wound management applications [21] However, transforming MSC-derived EV treatments from a clinical experiment into a mature medical commodity still faces many challenges in terms of controlling their identity, donor-related issues (variability and safety), quantity, size, purity, and fundamentally ensuring their scalability and cost-effectiveness [22,23].

Recent studies have demonstrated that EVs from different non-human and non-mammalian sources are characterized by morphological similarities but have different compositions and functions, displaying abilities as therapeutic effectors in wound healing [24,25]. Among these, edible plants represent an abundant natural source of EVs that is likely to allow for high-yield and large-scale EV production [25,26,27], Plant-derived EVs, in particular plant-derived nanovesicles (PDNVs), can be isolated directly from plant parts and tissues (fruits, roots, seeds, leaves, etc.) and juice, avoiding cell culture and expansion, thus significantly reducing the time and costs of the upstream production process typical of human cell-derived materials. To leverage this advantage of PDNVs, the entire isolation process needs to be optimized for efficient and high quality processing of large volumes of starting material. Studies pioneering the use of PDNVs used research-grade methodologies such as density gradient ultracentrifugation [28], a technique with important volume limitations and which is very time- and effort-intensive. Several techniques for the large-scale isolation of EVs that have already been implemented for downstream processing of human cell conditioned media include tangential flow filtration (TFF), size-exclusion chromatography (SEC), and microfiltration [6,29], but the application of these techniques on PDNVs is still in its infancy [30,31].

Another relevant aspect of PDNVs is that, being part of the human diet, they are likely non-toxic and non-immunogenic due to oral tolerance in most of the human population [32]. Recent research has shown that EVs present in food may physiologically interact with human metabolism [33]. It has also been demonstrated that edible plant-derived EVs induce expression of anti-inflammatory cytokine genes and antioxidant molecules that contribute to maintaining intestinal homeostasis [33]. Overall, EVs from plants exert natural beneficial effects on human health with potential therapeutic activities such as anti-tumor and anti-inflammatory effects, and no adverse effects have been reported [34,35,36]. Therefore, plant-derived EVs represent a good therapeutic candidate for topical wound treatment [24,25,26,34,36,37].

Accurately evaluating therapeutic efficacy on wound healing is imperative in clinical practice for managing chronic wounds in conditions where the natural physiological healing process is compromised. Diabetes mellitus is a primary contributor to impaired angiogenesis resulting in diminished wound healing capacity [15,38]. In diabetic patients, pathophysiological mechanisms involving cellular dysfunction, prolonged inflammation, hypoxia, neuropathy, deficient angiogenesis, and impaired neo-vascularization undermine the innate healing process [39,40]. Diabetic wounds present a worldwide medical challenge and are associated with a considerable socioeconomic burden. Further research into novel treatment modalities is necessary to enhance outcomes for this vulnerable patient population. At the same time, given the high and increasing prevalence of diabetes, high-value therapies include those which are cost-efficient and sustainable.

In the present study, we aimed to investigate the regenerative properties of EVs derived from from oranges (Citrus sinensis) in the context of skin wound healing in in vitro models as well as in vivo, in normal and streptozotocin-induced diabetic mice. We compared and evaluated the suitability of tangential flow filtration (TFF)- versus ultracentrifugation (UC)-based EV isolation from orange juice to implement the downstream production processes suitable for oEV scaleup. We also evaluated oEV formulations with commonly used hydrogels, supporting their use for topical applications.

## 2. Materials and Methods

### 2.1. Isolation of oEVs

oEVs were isolated from freshly squeezed orange juice processed as previously described [41]. We used oranges belonging to the species Citrus sinensis, type Tarocco, that were produced in Sicily, Italy and collected during the winter season from 2021 to 2023. UC-oEVs were isolated with an Optima L-90K ultracentrifuge, rotor 45 Ti, and polycarbonate tubes (Beckman Coulter, Milan, Italy) by two sequential centrifugation steps, first at 10,000× *g* for 1 h at +4 °C, and then at 100,000× *g* for 2 h at +4 °C. The pellets were re-suspended in saline solution (NaCl 0.9%, B. Braun, Melsungen, Germany) with 1% *v*/*v* DMSO (Sigma-Aldrich, Merck, Darmstadt, Germany), sterilized by filtration with 0.22 µm Millex syringe filters (Millipore, Merck, Darmstadt, Germany), and stored at −80 °C for further experiments. TFF-oEVs were isolated with a benchtop TFF system (CM500SE, Pall, Dreieich, Germany) equipped with a Centramate cassette (Pall) with a 100 kDa cut-off. Five liters of orange juice were clarified by filtration with 0.45 µm depth filters (Pall) then concentrated 20X and consequently diafiltrated 7 times with the TFF system. A saline solution (B. Braun) at pH 3 was used as a diafiltration buffer. Collected oEVs were sterilized by filtration with a 0.22 µm sterilizing grade filter (Pall), added to 1% *v*/*v* DMSO, and stored at −80 °C for further experiments. Wherever “UC” or “TFF” is not specified, the term “oEVs” is used for referring to UC-oEVs. Unless specified, all the in vitro and in vivo experiments were performed with UC-oEVs.

### 2.2. Characterization of oEVs

#### 2.2.1. Size, Concentration, and Zeta Potential

Size, concentration, and Z-potential measurements were performed as previously described [41]. Nanoparticle tracking analysis (NTA) was performed using the ZetaView TWIN Laser System with Zeta View software version 8.05.14 SP7 (ParticleMetrix, Inning am Ammersee, Germany). For analysis, oEVs were diluted at ratios ranging from 1:2000 to 1:10,000 in 5–10 mL of saline solution (0.9% NaCl, B. Braun, Melsungen, Germany) that had been filtered through 0.1 μm membranes (Millex, Millipore, Merck, Darmstadt, Germany).

The pH of oEVs was determined using an Orion Star A111 benchtop pH meter (Thermo Fisher Scientific, Waltham, MA, USA), with three measurements taken for each sample.

#### 2.2.2. Transmission Electron Microscopy

Transmission electron microscopy (TEM) was used to evaluate oEV structure and integrity. Sample preparation followed previously established protocols [41], with specimens examined using a Jeol JEM 1400 Flash electron microscope (Jeol, Tokyo, Japan). The preparation process involved placing oEVs on 200 mesh nickel formvar carbon-coated grids (Electron Microscopy Science, Hatfield, PA, USA) for 20 min to allow adhesion. The specimens were then treated with a solution of 2.5% glutaraldehyde containing 2% sucrose, followed by distilled water rinses. To complete the preparation, negative staining was performed using Nano-Van and Nano-W (Nanoprobes, Yaphank, NY, USA). Three independent experiments were conducted for each sample.

#### 2.2.3. Proteomic Analysis

For proteomic analysis, oEVs were concentrated by centrifugation. Briefly, oEVs were thawed, diluted in sterile water for diluting and washing salts, transferred in Amicon Ultra-15 centrifugal filter (Merck Millipore, Darmstadt, Germany), and centrifuged at 4500 rcf for 20 min at +4 °C. Proteomic analysis was performed by Gem For Lab (Caluso, Italy) following internal procedures. Briefly, for the 2-DE assay, proteins were denatured in urea- and thiourea-based buffer, loaded onto a pH 3–10 non-linear gradient IPG strip and separated at 20 °C on an Ettan IPGphor system. SDS-PAGE was performed on 12.5% polyacrylamide gels in a PerfectBlue Dual gel System, at 16 °C. 2-DE gels were stained with SYPROTM instructions and subsequently scanned by the CCD camera Chemidoc Imaging System. For SDS-Page assay, proteins were separated by molecular weight using Mini-Protean II apparatus (Bio-Rad, Hercules, CA, USA) equipped with Precast gel SDS 12% (Bio-Rad). Samples were prepared by addition of Laemmli Buffer (Bio-Rad), mixed and boiled at 95 °C for 5 min before loading into the wells. The gel was stained in Phast Gel Blue R working solution overnight, followed by 4 destaining steps. The selected band was excised from the gel, destained overnight, washed twice in 25 mM ammonium bicarbonate, twice in acetonitrile and dried. The gel fragment was reswollen in 100 mM ammonium bicarbonate containing 6 g of modified porcine trypsin and digested overnight at 37 °C. Peptides were extracted by sonication and analyzed by LC-MS/MS by a Q-TOF mass spectrometer. LC-MS/MS data were fed to the Mascot MS/MS Ions search algorithm for searching against the NCBIprot database, taxonomy: Viridiplantae (Green Plants). Mass tolerance for the monoisotopic peak masses was set to 10 ppm (parent ion) or 10 ppm (fragments), while the maximum number of missed cleavages was set to 2. In Mascot Search Results, best candidates (defined as “protein hits”) for each database search were listed according to their protein scores. Protein scores were derived from ions scores (defined as −10*Log(P), where P is the probability that the observed match is a random event) as a no probabilistic basis for ranking protein hits.

#### 2.2.4. Protein Quantification and Western Blot Analysis

For total protein quantification and Western blot analysis, oEVs were homogenized in RIPA buffer containing 1% *v*/*v* protease and phosphatase inhibitors and phenylmethylsulphonyl fluoride (PMSF). All products were acquired from Merck Millipore (Darmstadt, Germany). Protein concentration was determined using bicinchoninic acid (BCA) protein assay (Pierce, Thermo Fisher Scientific, Waltham, MA, USA) according to the manufacturer’s protocol as previously outlined [10]. Protein lysates were subsequently combined with Laemmli buffer (Bio-Rad) containing β-mercaptoethanol (Merck Millipore) and denatured at 95 °C for 5 min in a thermomixer. After cooling on ice, samples were loaded onto 4–20% Criterion TGX Stain-Free Precast gels (Bio-Rad) and transferred to nitrocellulose membranes (Bio-Rad). Membranes were blocked using EveryBlot Blocking buffer (Bio-Rad) for 10 min at room temperature, followed by overnight incubation with primary antibodies at +4 °C. All primary antibodies were polyclonal, rabbit-derived antibodies against Arabidopsis thaliana proteins, purchased from Phyto Ab (San Jose, CA, USA) and diluted 1:1000 in EveryBlot Blocking Buffer (Bio-Rad). A complete list of primary antibodies can be found in Supplementary Materials Table S1. Following overnight incubation, membranes were washed three times with PBS-Tween and incubated with species-specific, horseradish peroxidase (HRP)-conjugated Goat anti-rabbit secondary antibodies (1:10,000, Pierce, Thermo Fisher Scientific) for two hours at room temperature. Membranes were then washed three times with PBS-Tween. Chemiluminescent signals were developed using ECL substrate (Bio-Rad) and captured using the ChemiDoc™ MP Imaging System (Bio-Rad).

#### 2.2.5. RNA Quantification and Sequencing

Total RNA was extracted utilizing the miRNeasy mini kit from Qiagen (Hilden, Germany) according to the manufacturer’s protocol. The RNA was subsequently eluted in Ambion nuclease-free water (Thermo Fisher Scientific). RNA concentration was determined by measuring absorbance at 260 nm using a mySPEC spectrophotometer (VWR, Radnor, PA, USA). All samples were stored at –80 °C pending further analysis. RNA sequencing was performed by Dr. Francesca Anselmi and colleagues from the laboratory of Professor Oliviero (Department of Life Sciences and Systems Biology, University of Turin, Italy) according to validated internal procedures. The NEBNext^®^ Small RNA Library Prep (NEB) kit was used for small RNA. No ribosomal RNA depletion was performed for long RNA. Libraries were prepared using the TruSeq RNA Sample Prep Kit (Illumina, San Diego, CA, USA) following the manufacturer’s instructions. Libraries were sequenced on Illumina NextSeq 1000 (Illumina).

After quality controls (performed with FastQC v0.11.2 [42], sequencing reads were processed with fastp [43] to perform quality and adapter trimming. Trimmed sequences coming from long RNA libraries were aligned to the Csi valencia 1.0 genome assembly (accession number GCF_000317415.1) using STAR v2.7.1a51 with options: –outFilterMultimapNmax 10 –outFilterMultimapScoreRange 1 –outFilterMismatchNmax 999 –outFilterMismatchNoverLmax 0.04. Gene expression levels were determined with feature Counts v1.6.1 (https://subread.sourceforge.net/, accessed on 15 July 2024, options: -t exon -g gene_name) using the gene annotation associated to GCF_000317415.1 Csi_valencia_1.0. Reads that mapped to multiple locations were not included in the quantification process. Trimmed sequences coming from small RNA libraries were deduplicated using umi_tools [44] and analyzed using miRDeep2 [45] with csi miRabse sequence (version 22.1) as reference. Novel miRNAs were manually inspected using BLAST (2.16.0) against the NCBI nr/nt database in July 2024. Sequence and target similarity analysis of human and citrus miRNA sequences was performed using miRAl [46]. The mature sequence of the top expressed known and novel Citrus Sinensis (csi) miRNAs were aligned to the sequence of known human (hsa) miRbase miRNAs exploiting a modified version of the Needleman-Wunsch algorithm, where the match (mismatch) was assigned a greater score (penalty) in the substitution matrix. The seed was considered to be formed by nucleotides from 2 to 7 (6mer seed). For each csi miRNA, the best matching has-miRNA was identified and then the alignment was manually inspected filtering out cases in which the seed did not align properly. Then the overlap between the predicted target of each candidate mimicking miRNA and his human counterpart was evaluated by downloading 3′UTR from all Matched Annotation from NCBI and EMBL-EBI (MANE) transcripts of human protein coding from EN-SEMBL biomart and removing those without associated gene names. Then miRNA target prediction was performed using miRmap [47]. The top 100 genes for each miRNA were defined as putative targets; the same analysis was repeated for the hsa-miRNA possibly mimicking humans according to sequence alignment. The overlap between the predicted target of each candidate mimicking miRNA and his human counterpart was evaluated using Fisher’s exact test, Gene Ontology analysis, performed using miRphatDB v2 [48] for hsa miRNAs and clusterProfiler [49] on predicted target for csi miRNAs, using GO annotation retrieved in July 2024.

RNA sequencing was performed on six UC-oEV preparations and four TFF-oEV preparations run in triplicate. The comparison of the expression profiles was limited to the four pairs of UC- and TFF-oEV preparations obtained from the same orange juice batches. Heatmap of the top 50 mRNA more represented in oEVs was performed with Heatmapper [50]. Different oEV preparations were clustered with the Average Linkage method and Pearson distance measurement method.

#### 2.2.6. Raman Spectroscopy

For Raman analysis, 300 µL of oEVs were placed in a quartz cuvette and analyzed with a portable spectroscope Cora 100 (Anton Paar, Graz, Austria) equipped with a 785 nm laser. The presented spectra resulted from the average of three consecutive acquisitions performed on each sample.

#### 2.2.7. Lipidomic Analysis

For lipidomic analysis, oEVs were diluted 1:3 with sterile water and centrifugated at 100,000× *g* for 2 h. Pellets were resuspended in sterile water, frozen at −80 °C, and sent to Lipotype (Dresden, Germany) to perform the lipidomic analysis, following internal procedures. Briefly, lipids were extracted using chloroform and methanol [51]. For each oEV batch, three samples underwent analysis. Samples were spiked with Lipotype’s lipid class-specific internal standards prior to extraction. Following drying and reconstitution in MS acquisition mixture, the lipid extracts were analyzed using mass spectrometry. Mass spectra were collected using a hybrid quadrupole/Orbitrap mass spectrometer with an automated nano-flow electrospray ion source in both positive and negative ion modes. Lipid identification was performed on unprocessed (*.raw format) mass spectra using LipotypeXplorer [52]. In MS-only mode, identification relied on the molecular masses of intact molecules, while MSMS mode utilized both intact masses and fragment masses from collision-induced fragmentation. Lipid identifications underwent filtering based on mass accuracy, occupation threshold, noise, and background before normalization and statistical analysis. Lipid class-specific internal standards’ intensity enabled lipid quantification, with identified lipid molecules quantified through normalization to these standards. The total amount of each lipid class was calculated by summing the amounts (in pmoles) of individual lipid molecules (species or subspecies) within that class.

#### 2.2.8. Metabolomic Analysis

For metabolomic analysis, UC- and TFF-oEV volumes were adjusted to reach the same EV concentration in all samples. Metabolomic analysis was performed by Fondazione Edmund Mach (San Michele all’Adige, Italy) following internal procedures. Different classes of metabolites known to be present in citrus fruits were analyzed, including amino acids, flavonoids, terpenes, alcohols, aromatic compounds, acids, vitamins, carotenoids, total polyphenols, and total oxygen radical absorbance capacity (ORAC). Only metabolites that were present in detectable amounts are shown in the results. Four UC- and four TFF-oEV samples were analyzed.

### 2.3. In Vitro Cell Migration Assay

Cell migration assays were conducted on two cell types: Human dermal Microvascular Endothelial Cells (HMEC-1) from ATCC (Manassas, VA, USA) and the HaCaT keratinocyte cell line from Professor Vincenzo Calautti’s laboratory (University of Turin, Italy). The cells were cultivated in appropriate media—MCDB131 medium for HMEC-1 and DMEM for HaCaT—both supplemented with 10% fetal bovine serum at 37 °C in a 5% CO_2_ environment. Approximately 20 × 10^3^ cells were placed in each well of 24-well plates. Upon reaching confluence, two perpendicular scratches were made using a sterile tip to simulate a wound. Images were captured with a Leica camera before treatment. The cells then received one of several treatments: DMEM alone (control group), UC-oEVs at varying concentrations (10,000, 50,000, or 100,000 oEV/cell in DMEM), or 0.1 µg/mL EGF in DMEM as a positive control. Cell migration was assessed after 24 h for HMEC-1 and 48 h for HaCaT cells. The wound areas were measured using ImageJ 1.49v software. Three independent experiments were performed in triplicate. Results were calculated as migration percentages, with the pre-treatment wound area representing 100% and the cell-occupied area at the designated time points measured accordingly.

### 2.4. In Vitro Tube Formation Assay

The tube formation assay was conducted using HMEC-1 endothelial cells. These cells were placed at a concentration of 20,000 cells per well in a 24-well plate that had been previously coated with growth factor-reduced Matrigel (Corning, Tewksbury, MA, USA) under the following experimental conditions: DMEM alone (untreated cells, NT), UC-oEVs or TFF-oEVs at different doses (10,000, 30,000, 50,000, 75,000, or 100,000 oEV/cell in DMEM), or 10 ng/mL VEGF (Merck Millipore) in DMEM (Euroclone, Pero, Italy) as a positive control. After 24 h, to determine the development of capillary-like structures, wells were photographed using a Leica digital camera coupled to a Nikon-inverted microscope (10X). For each well, pictures were taken in five random fields. Automated image analysis was conducted using the Angiogenesis Analyzer tool within ImageJ 1.49v software. Results from three experiments, each performed in triplicate, are presented as the total length of capillary-like structures.

### 2.5. In Vitro Proliferation Assay

Cell proliferation was evaluated by tracing the integration of 5-bromo-2-deoxyuridine (BrdU) into the cellular DNA. The assay was performed on endothelial cells HMEC-1. Cells were seeded at the density of 2000 cells/well in 96-well plates with their culture medium and left to adhere. Once cells are attached, the culture medium is substituted with DMEM alone (starvation medium), and cells are left overnight in a CO_2_ incubator. Then, the plate is closed in a hypoxic chamber filled with the following mixture of gas: 5% CO_2_, 1% O2, 94% N. The hypoxic chamber is placed in a CO_2_ incubator for 24 h. Hen, the plate is removed from the hypoxic chamber, and cells are treated with the following conditions: DMEM alone (untreated cells, NT), UC-oEVs at different doses (10,000, 50,000, or 100,000 oEV/cell in DMEM), or 0.1 µg/mL EGF (Merck Millipore) in DMEM (Euroclone) as a positive control. Each condition is performed in quadruplicate. Then 10 µL of BrdU labeling solution (Roche, Basel, Switzerland) is added to each well and the plate is incubated overnight. The cells were treated with the stimuli for 24 h and with BrdU for 16 h. The ELISA assay was developed following the BrdU ELISA kit (Roche, Basel, Switzerland) manufacturer’s instructions. Absorbance measurements were obtained using an ELISA reader (Bio-Rad, Hercules, CA, USA). The experiments were conducted in triplicate, with results presented as a relative proliferation index compared to the control group of untreated cells (NT).

### 2.6. In Vitro Antioxidant Effect

#### 2.6.1. Antioxidant Effect on Hyperglycemia-Induced Protein Carbonylation

The antioxidant effect of oEVs was tested on Human microvascular endothelial cells (HMEC) cultured in hyperglycemic conditions to induce the production of free radicals with consequent protein carbonylation. Immortalized human dermal microvascular endothelial cells (HMEC) were created by using simian virus 40 immortalization of primary cells [53,54]. Control HMEC cells were cultured in Endothelial Basal Medium (EBM, Lonza) containing 5.6 mM glucose. High glucose and high mannitol media were created by adding either 28 mM α-D-glucose or 28 mM mannitol (Sigma Aldrich) to the EBM. The hyperglycemic (HG) model was established by continuously culturing HMEC in high glucose-EBM for 7 days. The intermittent hyperglycemic (INT HG) model alternated between high glucose-EBM and normal glucose-EBM every 48 h for 7 days. On day 5, HMEC cells were plated at a density of 10,000 cells per well in 8-well chamber slides with appropriate media. After adhering, the medium was replaced with DMEM (Euroclone) with or without 28 mM glucose supplementation and 10% ultra-centrifuged FBS. Cells were treated with oEVs at a ratio of 10,000 EVs per cell. On day 7, cells were washed with PBS, fixed with ice-cold methanol, and protein carbonyls (markers of oxidative stress) were labeled using 2,4-dinitrophenylhydrazine (DNPH) from the OxyICC kit (Merck Millipore) according to manufacturer’s protocol. DNPH was detected using a peroxidase-conjugated antibody with streptavidin substrate. Nuclei were counterstained with DAPI. All reagents came from the OxyICC kit. Immunodetection was performed using a fluorescent confocal microscope (Zeiss, Oberkochen, Germany) at 60× magnification. Five random fields were photographed per well. Fluorescence intensity was measured using ImageJ 1.49v software and Corrected Total Cell Fluorescence (CTCF) was calculated as previously described [55,56]. Results are presented as fold change compared to control (untreated cells). The experiment was performed in triplicate.

#### 2.6.2. Evaluation of Zonulin-1 Expression by Immunofluorescence

The expression of Zonulin-1 (ZO-1) protein was assessed on endothelial cells HMEC-1. Cells were seeded in 8-well Lab-Tek Chamber Slides (Thermo Fisher Scientific) at 5000 cells per well. Upon reaching 90–100% confluence, the cultures were injured using Cytomix, a combination of human IL-1β, TNF-α, and IFN-γ (25 ng/mL). oEVs were introduced concurrently with Cytomix at concentrations of 5000, 25,000, and 50,000 EV/cell. Following a 24-h incubation period, the cell monolayer was rinsed twice with PBS and fixed using 4% paraformaldehyde for 10 min. The cells were subsequently washed three times with PBS for 5 min and permeabilized with 0.2% Triton X-100 for 5 min. After three additional PBS washes, the slides were blocked with 1% BSA for 30 min at room temperature. For immunostaining, slides were incubated with ZO-1 rabbit anti-human primary antibody (Invitrogen, Thermo Fisher Scientific) at 7.5 µg/mL for 1 h at room temperature. Following three PBS washes, the slides were treated with Cy3™ goat anti-rabbit IgG (H + L) secondary antibody (Invitrogen, Thermo Fisher Scientific) for 1 h at room temperature. The slides were then washed with PBS, air-dried, and mounted using SlowFade™ Diamond Antifade Mountant containing DAPI (Invitrogen, Thermo Fisher Scientific). Images were captured using a confocal microscope Axiovert 200M equipped with LSM5 Pascal (Zeiss) with a 40×/1.3 NA oil immersion objective and acquired with a resolution of 0.44 × 0.44 μm. The fluorescent signal of ZO-1 staining was quantified with ImageJ software as Mean Gray Value, the sum of the gray values of all the pixels in the image divided by the number of pixels. For each well was estimated the Mean Gray Value of 3–5 images and the mean value was utilized for statistical analysis. Three experiments were done in duplicate.

#### 2.6.3. Antioxidant Effect on Cytokine-Stimulated Endothelial Cells

HMEC-1 cells were seeded in 24-well plates (Euroclone) at 90,000 cells per well. Upon reaching 90–100% confluence, the culture was subjected to injury using Cytomix, a combination of human IL-1β, TNF-α, and IFN-γ (25 ng/mL). After 6 h of exposure, oEVs were introduced at concentrations of 5000, 25,000, and 50,000 EV/cell. Following a 24-h incubation period, the supernatant was extracted, and cells were collected using TRY-EDTA (Merck Millipore). The cell suspension was then centrifuged at 1100 rpm for 7 min to obtain cell pellets for subsequent processing.

#### 2.6.4. Total and Mitochondrial (mt)ROS

We processed 1 × 10^5^ cells by washing them with PBS and gently scraping them for detachment. 50 µL aliquot of cells, was sonicated for cellular protein measurement. The remaining cell sample underwent a 30-min treatment at 37 °C with 5µM of either CM-H2DCFDA (Thermo Fisher Scientific) to measure total reactive oxygen species (ROS), or with 5µM MitoSOX (Thermo Fisher Scientific) to measure mitochondrial ROS. Both are ROS-sensitive fluorescent probes. We then converted the relative fluorescence units (RFUs) into nanomoles ROS/mg proteins using a calibration curve created with H_2_O_2_ serial dilutions.

#### 2.6.5. Lipoperoxidation and Protein Oxidation

The malonyl dialdehyde (MDA), an index of lipid peroxidation, was quantitated on 1 × 10^5^ cells using the Lipid Peroxidation (MDA) Assay Kit (Abcam, Cambridge, UK), as per manufacture’s protocol. Results were expressed as nmoles/mg total proteins. To evaluate protein oxidation, 1 × 10^5^ cells were lysed and the amount of carbonylated proteins was measured on 100 µg whole cell extract with Protein Carbonyl Content Assay Kit (Abcam). Results were expressed as nmoles/mg total proteins.

#### 2.6.6. Glutathione Measurement

For protein precipitation, 1 × 10^5^ cells were washed with 480 µL PBS, then treated with 120 µL of 6.5% *w*/*v* 5-sulfosalicylic acid. Samples were ice-incubated for 1 h and centrifuged at 13,000× *g* for 15 min at +4 °C. Total glutathione measurement was performed on 20 µL of lysate using a reaction mixture containing 20 µL stock buffer (143 mM NaH_2_PO_4_ and 63 mM EDTA, pH 7.4), 200 µL daily reagent (10 mM 5,5′dithiobis-2-nitrobenzoic acid and 2 mM NADPH in stock buffer), and 40 µL glutathione reductase (8.5 U/mL). To measure oxidized glutathione (GSSG), GSH was first derivatized with 2-vinylpyridine (2VP) by adding 10 µL of 2VP to 200 µL of lysate and shaking for 1 h at room temperature. Glutathione was then measured in 40 µL of this sample using the procedure described above. A Synergy HT Multi-Mode Microplate Reader (BioTek Instruments, Winooski, VT, USA) was used to monitor the reaction kinetically for 5 min by measuring absorbance at 415 nm. All measurements were performed in triplicate with results expressed as nmoles glutathione/min/mg cell proteins. Reduced glutathione (GSH) values were calculated by subtracting GSSG from total glutathione measurements.

#### 2.6.7. Antioxidant Enzymes

Glucose 6-phosphate dehydrogenase (G6PD) activity was assessed in a specific protocol. Cells were initially washed using fresh medium, separated using trypsin/EDTA, and then resuspended at a concentration of 0.1 × 10^6^ cells/mL in 0.1 M Tris/0.5 mM EDTA at pH 8.0. The suspension underwent sonication on ice with two 10-s bursts to create a cell lysate. This lysate was then enhanced with 10 mM MgCl2 and 0.25 mM NADP+. To initiate the reaction at 37 °C, 6-phosphogluconate (0.6 mM) was added, with or without glucose 6-phosphate (0.6 mM). The resulting enzymatic activity was quantified spectrophotometrically by measuring the absorbance increase per minute at 340 nm using a Synergy HTX 96-well microplate reader. A first measurement was performed by adding to the assay system a saturating amount of both 6-phosphogluconate and glucose 6-phosphate: the rate of NADP+ reduction was the result of both G6PD and 6PGD activities. A second assay was conducted using only 6-phosphogluconate as substrate, which measured 6PGD activity exclusively. G6PD activity was calculated by subtracting the second assay rate from the first assay rate [57]. Reaction kinetics-maintained linearity throughout the 5-min observation period. Enzymatic activity was quantified as nmoles NADPH/min/mg cell proteins.

Cytosolic superoxide dismutase 1 (SOD1) and mitochondrial superoxide dismutase 2 (SOD2) activity measurements used 10 μg of cytosolic and mitochondrial proteins, following cytosol-mitochondrial separation as described in [58]. After resuspension in 100 µL PBS, samples were incubated with 50 μM xanthine, 5 U/mL xanthine oxidase, and 1 μg/mL oxidized cytochrome c for 5 min at 37 °C. The cytochrome c reduction rate inhibited by SO, was monitored for 5 min by measuring absorbance at 550 nm using a Packard microplate reader EL340 (Bio-Tek Instruments, Winooski, VT, USA). Results were expressed as nmoles reduced cytochrome c/min/mg cytosolic or mitochondrial proteins.

The activities of glutathione reductase (GR), glutathione peroxidase (GPX), and thioredoxin reductase (TrxR) were measured with the following commercial kits (all from Abcam): Glutathione Reductase (GR) Assay Kit, Glutathione Peroxidase Assay Kit, Thioredoxin Reductase Assay Kit, according to manufacturer’s instructions. Results were expressed as enzymatic units (U)/mg cell proteins, based on the titration curve of each kit.

### 2.7. Hydrogel Formulation and oEV Release

Hydrogel formulations were prepared as follows. Carboxymethylcellulose high viscosity (Sigma-Aldrich, St Louis, MO, USA) was dissolved in distilled water to 10 mg/mL, and chitosan (Sigma-Aldrich) was dissolved in acetic acid to 2%. All hydrogels were sterilized by autoclave before use and were used alone as negative control, or in formulation with UC-oEVs at different concentrations.

For measuring the release of oEVs from hydrogels over time, carboxymethylcellulose and chitosan hydrogels with or without oEVs at different doses (10^8^, 10^9^ and 10^10^ EVs), were kept in a 24-well plate in direct contact with saline solution. The saline solution was analyzed for the presence of EVs at time 0 (0) and after 30 min 1 h, 3 h, 6 h, 24 h, 48 h, 72 h. oEVs were quantified in the saline solution at each time point by using the NanoSight LM10 system (Malvern, Malvern, UK). The amount of EVs released by hydrogel was expressed as number of EV/mL. Three experiments were performed for each data set.

For evaluating the uptake of oEVs released from the hydrogels by target cells, HMEC-1 were treated with different doses of oEVs (10^8^, 10^9^ or 10^10^ EVs) fluorescently labeled with the fluorochrome PKH26 (Sigma-Aldrich) with or without carboxymethylcellulose hydrogel. The uptake of EVs in target cells was evaluated using flow cytometry (CytoFLEX flow cytometer, Beckman Coulter, Indianapolis, IN, USA) at multiple time intervals: 6, 24, 48, and 72 h. Each data set was derived from three independent experiments.

To evaluate the biological effects of oEVs released from the hydrogels, the tube formation assay (see Section 2.4 and the migration assay (see Section 2.3) were repeated. For the tube assay and the migration assay carboxymethylcellulose (H1) and chitosan gel (H2) were used. For both experiments, HMEC-1 were stimulated with oEVs alone (10^9^ EV/cell), hydrogel alone, or the combination of oEVs (10^9^ EV/cell) and hydrogel, for 24, 48, and 72 h. Three experiments were performed for each data set.

### 2.8. Wound Healing Mouse Models

All experiments on in vivo models were conducted by Artimmune SAS in an Artimmune’s laboratory by authorized technicians regularly employed by the company. Manipulations of animals were conducted gently to reduce stress to the minimum. All experiments were conducted in compliance with the Declaration of Helsinki and the guidelines of the French Ministry of Agriculture for experiments with laboratory animals (law 87-848). The research was performed in accordance with Animal Health guidelines, specifically following:

- Council Directive 2010/63/UE (22 September 2010) regarding the protection of animals used for scientific purposes, as well as French decree 2013-118 (1 February 2013) concerning animal protection.
- Artimmune/CNRS facility accreditation for experimentation (N° F45-234-6), for wound healing model project accreditation for experimentation (CLE-CECCO-2062 #34498), and approved by the Ethics Committee of Artimmune (22/12/2021).

Normal BALB/c or diabetic NOD.SCID (NGS) male mice (from Charles River Laboratories, Lyon, France) at 8 weeks of age were used for the experiments. Diabetes mellitus was induced in NGS mice by intraperitoneal injection of streptozotocin (STZ, 40 mg/kg, i.p. for 5 days), an antibiotic isolated from *Streptomycin achromogenes* that induces pancreatic β-cells destruction as previously described [59,60]. Blood glucose levels were measured on days 0, 5, 10, and 15 post injections, until day 20. Animals were treated after about 20 days from diabetes induction.

Minimal statistical number of 10 mice/group (total 80) were used.

For wound induction, mice were anesthetized with slight isoflurane anesthesia and back cutaneous hair was removed by electrical shaving. Mice were then depilated with Nair hair removal cream and cleaned with water. Then full-thickness skin wounds were created with a sterile 4 mm diameter biopsy punch (Stiefel, PMD Medical, France), four wounds per mouse. Mice were housed for the duration of the experiment in pathogen-free conditions using ventilated cages. Each animal was treated with 20 µL of hydrogel alone, carboxymethylcellulose high viscosity 10 mg/mL (Sigma- Aldrich) (H1) or chitosan hydrogel (H2), or hydrogel formulated with oEVs at different doses (10^9^, 10^10^, 10^11^ UC-oEVs). Untreated mice were used as negative controls (NT). Wound initiation day was designated as day 0. Animal monitoring occurred at specific intervals (days 0, 3, 7, and 10 for BALB/c mice; days 0, 3, 7, 10, and 14 for NGS mice), during which body weight was recorded and treatments were freshly applied to wounds. Daily wound documentation was performed using a Nikon D3500 digital camera. Wound measurements were conducted using ImageJ software, with closure percentage calculated as: (current closed wound size/initial wound size) × 100. The current closed wound size was determined by subtracting the current wound size from the initial wound size. Upon reaching the final timepoint, mice were euthanized, and back skin wound samples were collected for histological examination.

Back skin was fixed in 4% buffered formaldehyde (X1 Formal-fixed Fixateur, Thermo Fisher Scientific) for a minimum of 72 h, embedded in paraffin using LOGOS equipment for standard microscopic analysis (Leica microscope) and then stained with hematoxylin/eosin (H&E) staining. Total wound size, the size of an open wound, and epidermal thickness were measured with ImageJ software on H&E stained sections.

### 2.9. Statistical Analysis

Statistical analysis was conducted using GraphPad Prism 6.0 Demo (GraphPad, San Diego, CA, USA). The analytical method was selected based on group quantity: ANOVA was utilized for three or more groups, while *t*-tests were applied when comparing two groups. Results are presented as mean ± SD or SEM. Statistical significance was defined as *p* < 0.05, with significance levels indicated as follows: * *p* < 0.05, ** *p* < 0.01, *** *p* < 0.005, and **** *p* < 0.001. The designation “ns” indicates no statistical significance (*p* > 0.05).

## 3. Results

### 3.1. oEVs’ Characterization and Comparison of UC- and TFF-oEVs

Purification and characterization were performed according MISEV guidelines [5,6], specifically evaluating visual inspection, concentration (NTA), size, total protein, total RNA, pH, Zetapotential, Raman Spectroscopy fingerprints and morphology. Table 1 shows reproducibility of different batches produced by ultracentrifugation and TFF. The number in the sample name indicates the orange Juice batch.

**Figure 1:**
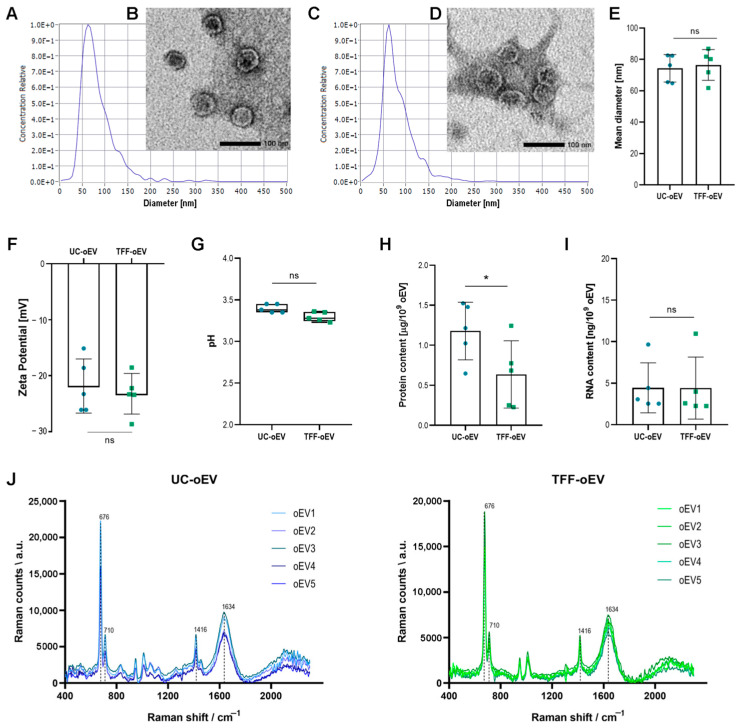
Characterization of UC-oEVs and TFF-oEVs. (**A**–**D**) Representative images of UC-oEVs and TFF-oEVs analyzed with Nanoparticle Tracking Analysis (NTA), and Transmission electron microscopy (TEM). Representative NTA (**A**) and TEM (**B**) of UC-oEVs and NTA (**C**) and TEM (**D**) of TFF-oEVs. TEM scale bar 100 nm. (**E**) NTA analysis of EV size, shown as mean diameter and expressed in nanometers (nm). (**F**) Zeta potential analysis expressed in milli volt (mV). (**G**) pH measurements. (**H**) Total protein content, expressed as µg of proteins in 10^9^ oEVs. (**I**) Total RNA content, expressed as ng of proteins in 10^9^ oEVs. (**J**) Raman spectra for UC-oEVs (**left**) and TFF-oEVs (**right**). All experiments were performed on *n* = 5 different oEV preparations. Data are represented as mean ± SD. ns: not statistically significant. * *p* < 0.05.

oEVs purified from orange juice by ultracentrifugation (UC-oEVs) and TFF (TFF-oEVs) were also compared. As seen in Figure 1, UC-oEVs and TFF-oEVs showed similar size and concentration, as detected by Z-view instrument (Figure 1A,C,E). Both oEV preparations displayed nanorange size with no statistical differences observed in the mean diameters, being 74.32 ± 8.75 nm for UC-oEVs and 76.48 ± 9.82 nm for TFF-oEVs (Figure 1D). The TEM inspection of the morphological aspect of UC-oEVs and TFF-oEVs allowed the observation of a distinct round membrane including a dense core (Figure 1B,D).

TEM confirmed the size in the range of 60 to 100 nm for both UC-oEVs and TFF-oEVs. The Zeta potential for both UC-oEVs and TFF-oEVs was similarly negative below −20 mV, 21.84 ± 4.84 mV and −23.23 ± 3.62 mV respectively (Figure 1F), and pH was acid, 3.40 ± 0.05 for UC-oEVs and 3.20 ± 0.06 for TFF-oEVs (Figure 1G). The concentration of proteins was statistically lower in TFF-oEVs preparation with respect to UC-oEVs, 0.64 ± 0.42 and 1.18 ± 0.36 µg/10^9^ oEVs respectively (Figure 1H), suggesting a better removal of contaminant proteins by TFF purified oEVs. Conversely, the RNA content was identical in UC-oEVs and TFF-oEVs, with 4.44 ± 3.01 and 4.40 ± 3.73 ng/10^9^ oEVs, respectively (Figure 1I). A comparison of Raman fingerprints of five different preparations of UC-oEVs and TFF-oEVs showed good reproducibility of independently purified oEVs prepared with both methods (Figure 1J). Raman measures of H_2_O and saline solution used as oEV suspension buffer did not show any signal, confirming that observable peaks are representative of the oEV content. All the oEV samples showed four main peaks at 676, 710, 1416, and 1634 cm^−1^.

These data demonstrated that typical EV characteristics prior described for UC-oEVs were maintained by TFF-oEVs, confirming that the TFF technique is appropriate for oEV isolation.

### 3.2. Molecular Characterization of oEV

**Figure 2:**
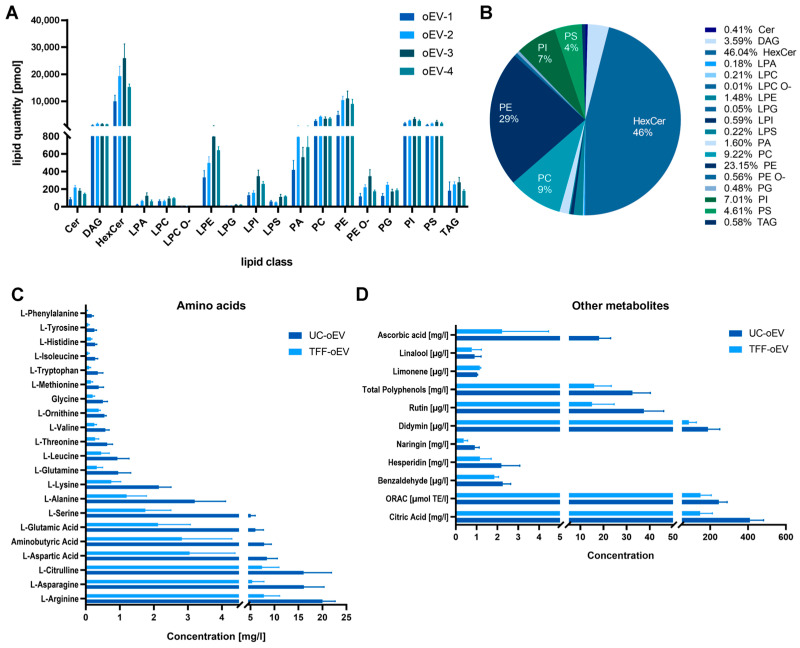
Lipidomic and metabolomic analysis of oEVs. (**A**,**B**) Lipidomic analysis of n = 4 different UC-oEV preparations. (**A**) Lipid quantity expressed in picomoles (pmol) for each lipid class, data are represented as mean ± SD. (**B**) Mean percentage of each lipid class on the total lipid quantity. Abbreviations: Cer, Ceramides; DAG, Diacylglycerols; HexCer, Hexosylceramides; LPA, Lyso-Phosphatidates; LPC, Lyso-Phosphatidylcholines, LPC O-, Ether-linked Lyso-Phosphatidylcholines; LPE, Lyso-Phosphatidylethanolamines; LPG, Lyso-Phosphatidylglycerols; LPI, Lyso-Phosphatidylinositols; LPS, Lyso-Phosphatidylserines; PA, Phosphatidates; PC, Phosphatidylcholines; PE, Phosphatidylethanolamines; PE O-, Ether-linked Phosphatidylethanolamines; PG, Phosphatidylglycerols; PI, Phosphatidylinositols; PS, Phosphatidylserines; TAG, Triacylglycerols. (**C**,**D**) Metabolomic analysis of *n* = 4 different UC- and TFF-oEV preparations. Data are represented as mean ± SEM. The histograms show the concentration of the amino acids (**C**), expressed as mg/L, and of the other metabolites (**D**), expressed as indicated in the *Y*-axis label, present in both oEV types. Abbreviations: ORAC, oxygen radical absorbance capacity.

The lipidomic analysis was performed on four different preparations of UC-oEVs. As shown in Figure 2A, the lipid quantification revealed a similar amount of the different lipid classes in the tested oEV preparations, indicating a good batch reproducibility of the lipid profile. The percentage distribution of different lipid classes indicates a prevalence of Hexosylceramides (HexCer, 46%), followed by Phosphatidylethanolamines (PE, 29%), Phosphatidylcholines (PC, 9%), Phosphatidylinositols (PI, 7%), and Phosphatidylserines (PS, 4%), as shown in Figure 2B.

Metabolomic analysis showed a generally higher amino acid concentration in UC-oEVs compared to TFF-oEVs. This result is consistent with the total protein concentration. However, the distribution profile of the individual amino acid is similar in the two oEV types (Figure 2C). The analysis of the other metabolites (Figure 2D) showed that the oxygen radical absorbance capacity (ORAC) was very high in both samples. Metabolites with potential antioxidant activity are highly expressed in oEVs, including ascorbic acid, one of the basic antioxidants regulating the level of reactive oxygen species (ROS) and the efficacy of other antioxidants. Several other molecules with antioxidant and anti-inflammatory properties are present including polyphenols, rutin, naringin, benzaldehyde, and citric acid, which is known to inhibit oxidation by scavenging reactive oxygen species (ROS) [61]. In addition, oEVs contain a high level of didymin which has been reported to retain, besides the antioxidant properties, a variety of pharmacological activities including anticancer, neuroprotective, hepatoprotective, and antinociceptive activities [62]. oEVs also contain hesperidin, a flavanone that exerts anti-inflammatory and antioxidant activities and retains neuroprotective properties [63].

Proteomic analysis was performed on four oEV preparations. 2-DE gels followed by MS analysis and Mascot identification gave a list of 11 proteins (Table A1) and the direct MS analysis followed by Mascot identification identified 35 proteins (Table A2). The two lists contained the same proteins. Some proteins belonged to plants of the genus Citrus, and most of them were from other plant genera. This is probably due to a poor annotation of plant databases, as proven by the names “unnamed protein” or “hypothetical protein”. To better investigate the type and function of identified proteins, UniProt database [64] was used for searching Protein IDs and the homologous for Citrus sinensis and Arabidopsis thaliana (the better-annotated plant organism). Identified proteins were grouped into classes based on their function or reconducted to a single protein type (when different subunits of the same protein were identified). The biological functions of each identified protein or protein class are described in Table 2. To confirm the results of the analysis, WB was performed on a selected panel of proteins on three UC- and TFF-oEV preparations. In addition, some proteins described in the literature (also listed in Table 1) were tested. ARP7, CHC1, and Hsp70-2 were detected in Citrus sinensis-derived EVs [65] and are usually present in stem cell-derived EVs. PDI5 was indicated as a putative marker of plant-derived EVs [66]. TET8 is a homolog of tetraspanins recognized as canonical markers of human stem cell-derived EVs and it was found in plant-derived EVs [27].

**Figure 3:**
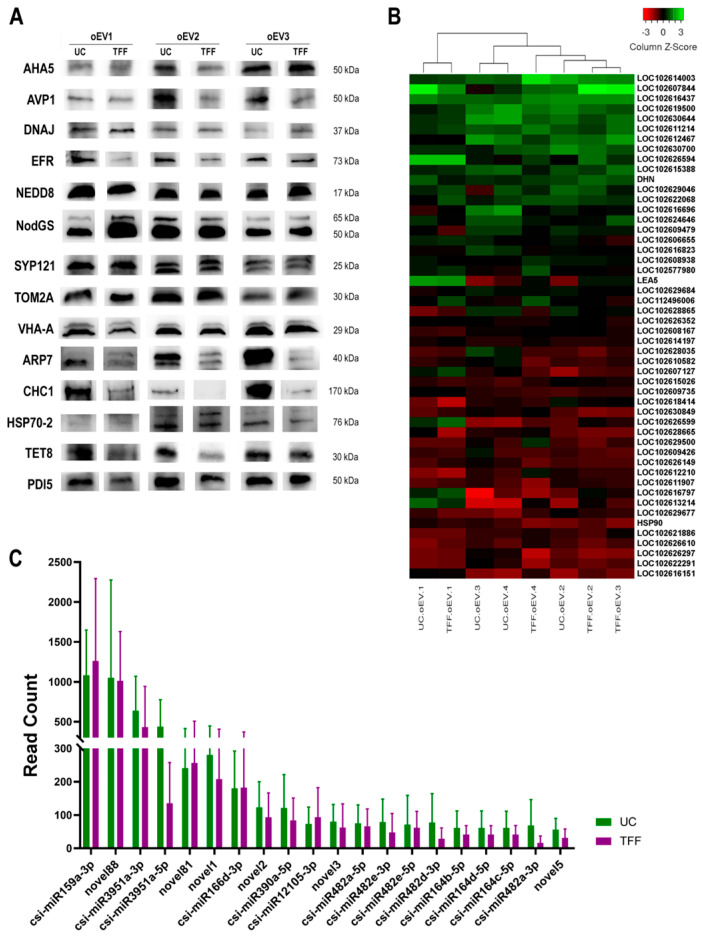
Proteomic analysis and RNA sequencing of oEVs. (**A**) Representative Western Blot analysis of n = 3 different UC- and TFF- oEV preparations. (**B**) Heatmap of the top 50 mRNA more represented in oEVs, clustering was applied on *n* = 4 different oEV preparations. (**C**) Histogram of the top 20 miRNA in UC- and TFF- oEV. Data are shown as mean ± SD of *n* = 4 different UC- and TFF-oEV preparations.

WB analysis showed that all proteins identified by the proteomic analysis were expressed in all samples (Figure 3A). No differences were observed between EVs isolated with UC and TFF, suggesting that the two isolation techniques do not select different subpopulations of EVs.

The analysis of long RNAs performed on the results of RNA sequencing showed an abundant presence of ribosomal RNA (rRNA) (Figure S1A) and the expression of 10,000 genes out of 28,000 genes annotated in the reference genome, with 18,600 genes showing at least one read in one sample and 15,000 showing, on average, more than one read per million (Figure S1B). The expression levels were similar for all genes, without subsets of genes overexpressed. These characteristics were compatible with cytoplasmic RNA.

The principal component analysis (PCA) of the long RNAs showed that 25% of the variance was associated with the supplier of the orange juice (PC1, Figure S1C), and the extraction method (UC or TFF) was not correlated with the principal components PC1 and PC2 (Figure S1D). Moreover, the expression profiles were similar in all tested samples, independently from the isolation method, as shown by the clustering analysis that does not cluster the samples based on the isolation method (UC or TFF) or the juice batch (1, 2, 3, 4) (Figure 3B). Noteworthy, among the most expressed genes there was LOC102619500, corresponding to the probable inactive poly [ADP-ribose] polymerase SRO5, LOC102577980, corresponding to actin-7, DHN, LEA5, HSP90, the heat shock protein 90, LOC102608938, corresponding to dnaJ protein homolog, LOC102624646, corresponding to thioredoxin H-type. The full results of long RNA sequencing are available in Supplementary Table S2.

The RNA sequencing analysis identified 83 miRNA, some of which were not described in miRbase database and were denominated *novel*. BLAST analysis on predicted novel miRNA showed that 37.9% were probably true miRNA (Figure S2A). Novel88 is classified as possible contaminant because it did not align on *C sinensis* genome, however, this may also be due to lacking in *C sinensis* genome annotations. A validation on *C sinensis* DNA should be performed to clarify this point. The PCA analysis did not show a variance correlated to the supplier of the orange juice (Figure S2B) or the extraction method (Figure S2C).

Similarly to mRNA, miRNA identified by RNA sequencing showed a comparable expression level in UN- and TFF-oEVs (Figure 3C). The mean read count was similar for all the top 20 miRNAs without significant differences between UC- and TFF-oEVs.

To better understand their function, the mature sequence of the top expressed known and novel Citrus Sinensis (csi) miRNAs were aligned to the sequence of known human (hsa) miRNAs. Interestingly, 12 miRNAs showed a high degree of similarity with human miRNAs. Results and the known functions of the hsa miRNA are shown in Table A3. Moreover, miRNA target prediction was performed on the 67 known and novel miRNAs showing at least 10 reads on average. When more than one target was predicted on a 3′UTR only the best one was retained. The top 100 genes for each miRNA were defined as putative targets and the same analysis was repeated for the hsa-miRNA possibly mimicking human miRNAs detected with the previous sequence alignment. Then the overlap between the predicted target of each candidate mimicking miRNA and his human counterpart was evaluated.

The analysis showed that the pairs of miRNAs csi-miR164a-5p and hsa-miR-5703, csi-miR3954 and hsa-miR-4520-3p, csi-miR166b-3p and hsa-miR-10399-3p, showed a significant predicted target overlap, another evidence that suggests a possible mimicking. The full list of predicted miRNA targets is available in Supplementary Table S3. Interestingly GO analysis predicted that hsa-miR-5703 was involved in tube development (306 hits, 1.35 × 10^−7^ *p*-value) and vasculature development (211 hits, 1.50 × 10^−5^ *p*-value) and hsa-miR-10399-3p was involved in cell adhesion (550 hits, 1.42 × 10^−4^ *p*-value).

Since no function predictions were available for csi miRNA, we carried out a prediction starting from predicted targets previously identified. The best prediction overall is for the miRNA novel73, the predicted function is homophilic cell adhesion via plasma membrane adhesion molecules (GO:0007156) and is very strong (adjusted *p* value < 3.2 × 10^−17^). Novel73 is a medium-expressed miRNA that has some similarities with cssat_11 satellite repeat. Other suggestive predictions are listed in Table 3.

### 3.3. oEVs Showed Pro-Regenerative Effects In Vitro

**Figure 4:**
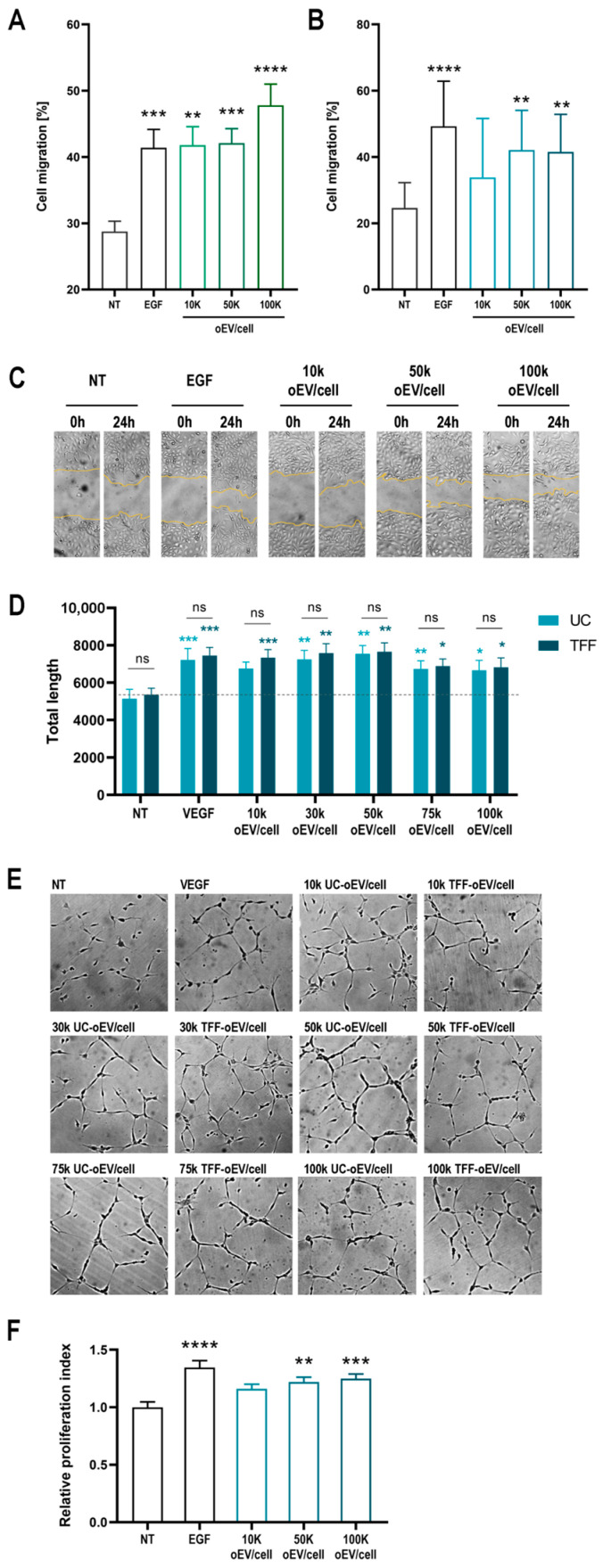
Pro-regenerative effects of oEVs in vitro. (**A**–**C**) Cell migration assay on endothelial cells (**A**), and keratinocytes (**B**), results are shown as the percentage of cell migration at respectively 24 or 48 h from the scratch. (**C**) Representative images of endothelial cells at 0 and 24 h from the scratch, the borders of the cell layer are highlighted by the yellow line. (**D**,**E**) Tube formation assay on endothelial cells stimulated with UC- or TFF-oEVs at different doses. (**D**) Results are shown as the mean total length of the capillary-like structures formed by HMEC seeded on Matrigel-coated plates. (**E**) Representative pictures of the capillary-like structures at 10× magnification. (**F**) BrdU proliferation assay on endothelial cells in hypoxic conditions, expressed as a ratio over untreated cells (NT). EGF and VEGF were used as positive controls. All histograms show the mean ± SEM from *n* = 4 experiments. The statistical significance was calculated by comparing all conditions vs. NT and in (**D**) UC-oEVs vs. TFF-oEVs. ns: not statistically significant, * *p* < 0.05, ** *p* < 0.01, *** *p* < 0.005, and **** *p* < 0.001.

To evaluate in vitro the wound healing properties of oEVs, a scratch test was performed on a monolayer of human endothelial cells and keratinocytes, then cell migration was measured. oEVs promoted significant cell migration of endothelial cells and keratinocytes, expressed as a percentage of cell migration, comparable with the migration induced by EGF used as positive control (Figure 4A,B). The Figure 4C is representative of endothelial cell migration after 24 h of stimulation with oEVs at different ratios (10,000, 50,000, and 100,000 oEV/cell) or with EGF. Since the effect on motility of endothelial cells after oEV stimulation was greater than that observed for keratinocytes, we explored in vitro a potential pro-angiogenic effect of oEVs. oEVs were found to promote the organization of endothelial cells plated on Matrigel in vessel-like structures with an efficiency comparable with VEGF used as positive pro-angiogenic control (Figure 4D). The pro-angiogenic efficacy measured as the length of capillary-like structures was comparable for UC-oEVs and TFF-oEVs. Figure 4E shows the typical organization of endothelial cells forming capillary-like structures. Moreover, oEVs at higher doses promoted also the proliferation of hypoxia-stimulated endothelial cells (Figure 4F).

### 3.4. oEVs Showed a Strong Antioxidant Effect In Vitro

Due to the high presence of antioxidants revealed by the metabolomic analysis, the antioxidant activity of oEVs was tested in various in vitro models.

**Figure 5:**
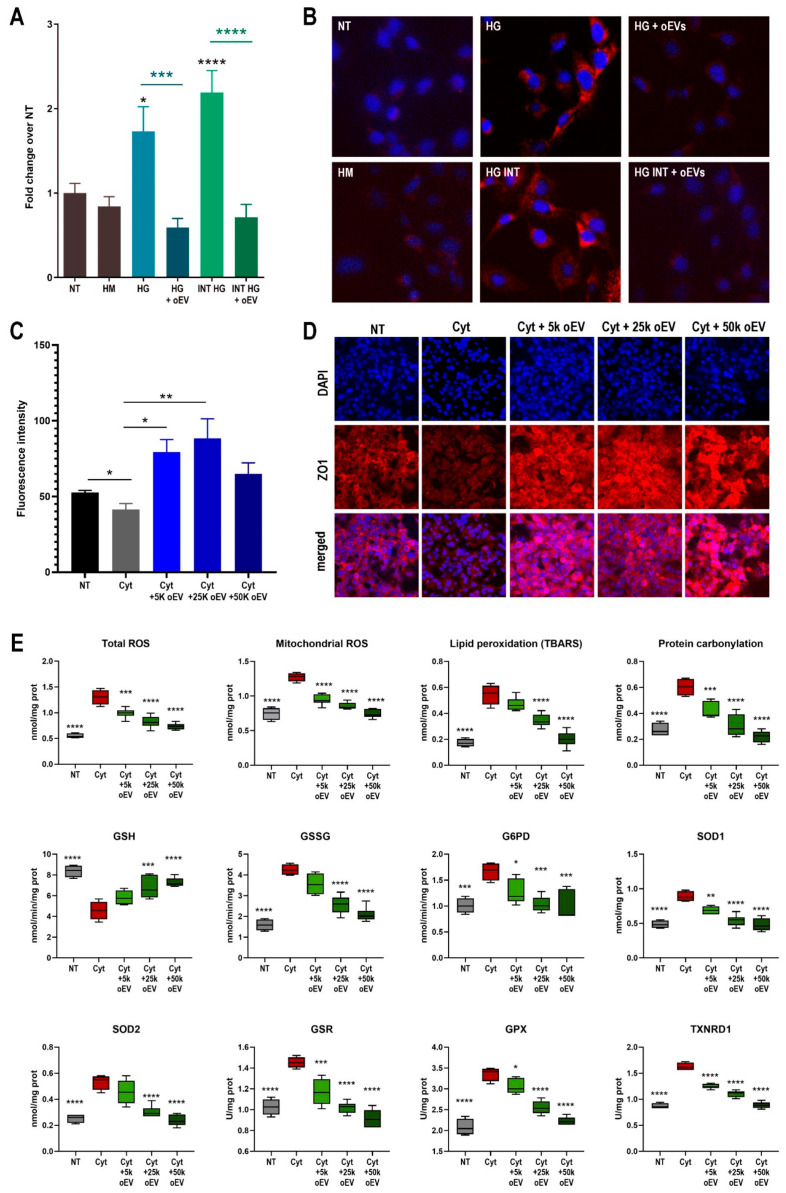
Antioxidant effects of oEVs. (**A**,**B**) Antioxidant effect of oEVs on protein carbonylation induced by culturing endothelial cells in constant (HG) or intermittent (INT-HG) hyperglycemic conditions. (**A**) The histogram shows the Corrected Total Cell Fluorescence (CTCF) fold change over untreated cells (NT), mean ± SEM. (**B**) Representative images of immunofluorescent staining of nuclei with DAPI (blue) and oxidized proteins with 2,4-DNPH (red). Pictures were taken by Zeiss fluorescent confocal microscope at 60× magnification. (**C**,**D**) oEVs induction of Zonulin 1 (ZO-1) expression in inflammatory conditions obtained by treating endothelial cells with a cytokine cocktail (Cyt). (**C**) The histogram shows the Fluorescence intensity, mean ± SEM. (**D**) Representative images of immunofluorescent staining of nuclei with DAPI (blue) and ZO-1 (red). Pictures were taken by Zeiss fluorescent confocal microscope at 43× magnification. (**E**) Antioxidant effects of oEVs on cytokine-stimulated endothelial cells. For each dataset, unit of measurement are expressed as indicated in the *Y*-axis labels. Abbreviations: ROS, Reactive Oxygen Species, TBARS, thiobarbituric acid reactive substances; GSH, reduced glutathione; GSSG, oxidized glutathione; G6PD, glucose 6-phosphate dehydrogenase; SOD1, superoxide dismutase 1; SOD2, superoxide dismutase 2; GSR, glutathione reductase; GPX, glutathione peroxidase; TXNRD1, thioredoxin reductase. *n* = 4 experiments were performed for each data set. * *p* < 0.05, ** *p* < 0.01, *** *p* < 0.005, and **** *p* < 0.001.

We cultured endothelial cells in constant or intermittent high glucose stimulation that mimics the in vivo conditions in diabetic patients and promotes the formation of free radicals leading to protein carbonylation. oEVs significantly reduced protein carbonylation induced by the hyperglycemic conditioning (Figure 5A,B).

Zonulin 1 (ZO-1) expression in endothelial cells counteracts the increased permeability in inflammatory conditions [67]. Inflammation was mimicked in vitro by stimulating endothelial cells with Cytomix (Cyt), a cocktail of pro-inflammatory cytokines, which reduce ZO-1 expression. oEVs showed to revert this effect by significantly increasing ZO-1 expression compared to endothelial cells after Cytomix stimulation (Figure 5C,D).

The Cytomix also induced a significant oxidation injury on endothelial cells as shown in Figure 5E. oEVs treatment showed a strong and dose-dependent antioxidant activity at multiple metabolic levels (Figure 5E). oEVs were shown to reduce both total ROS and mitochondrial ROS and to counteract ROS damages by reducing lipid peroxidation and protein carbonylation. They were also shown to increase reduced glutathione (GSH) and reduce oxidized glutathione (GSSG). Moreover, analyzing the activity of the antioxidant enzymes, in the presence of oEVs the activity of glucose 6-phosphate dehydrogenase (G6PD), cytosolic superoxide dismutase 1 (SOD1), mitochondrial superoxide dismutase 2 (SOD2), glutathione reductase (GR), glutathione peroxidase (GPX), and thioredoxin reductase (TrxR) were significantly reduced as well as ROS levels.

Although oEVs show robust antioxidant activity, the observed decrease in the activities of GR, GPx, and TrxR is not necessarily contradictory. In our model, oEV treatment reduces oxidative stress, as shown by lower total ROS, mitochondrial ROS, lipid peroxidation, and protein carbonylation. Under these conditions, cells may have a diminished requirement for high flux through the glutathione and thioredoxin antioxidant systems. Because GR/GPx/TrxR activities are, at least in part, responsive to oxidant burden and redox cycling demand, a reduction in ROS/peroxide load can translate into lower measured enzyme activity (i.e., less compensatory upregulation and less substrate-driven turnover).

### 3.5. The Formulation of oEVs with Hydrogels Promotes a Controlled Release of Functional oEVs over Time

**Figure 6:**
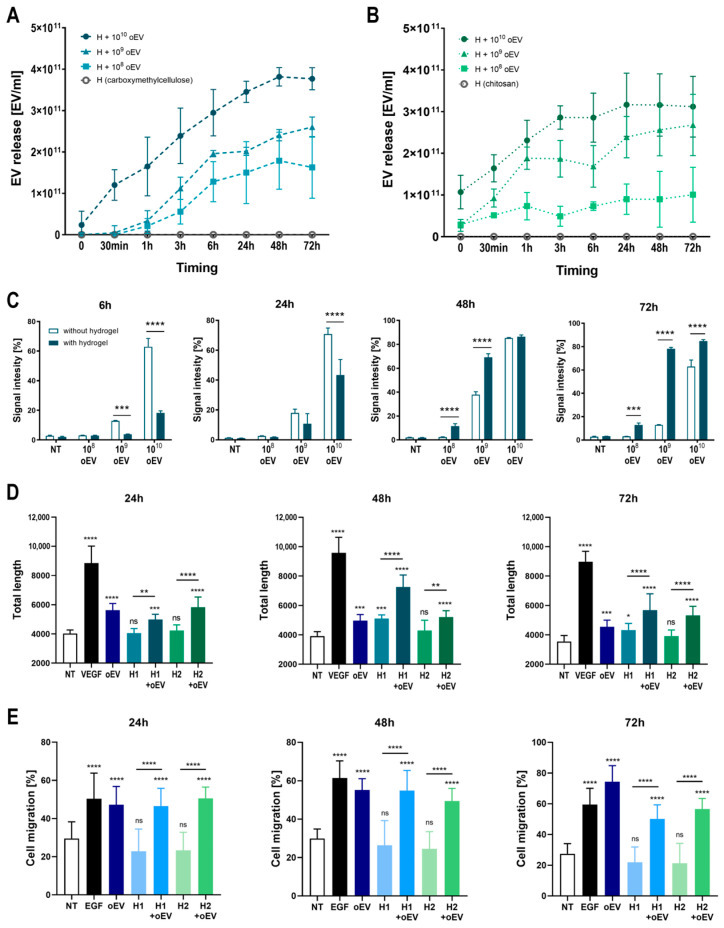
Formulation of oEVs with hydrogels. (**A**,**B**) Release of different doses (10^8^, 10^9^, 10^10^) of oEVs from carboxymethylcellulose hydrogel (**A**) or chitosan hydrogel (**B**) over time, from 30 min to 72 h. (**C**) Uptake of different doses of fluorescently labeled oEVs alone (oEV) or released from carboxymethylcellulose hydrogel (H + oEV) in endothelial cells, measured by flow cytometry at different time points: 6, 24, 48, and 72 h. (**D**) Tube formation assay on endothelial cells stimulated with oEV alone or oEV released from carboxymethylcellulose hydrogel (H1 + oEV) or chitosan hydrogel (H2 + oEV) after 24, 48, or 72 h. Results are shown as the mean total length of the capillary-like structures formed by HMEC seeded on Matrigel-coated plates. (**E**) Cell migration assay on endothelial cells stimulated with oEV alone or oEV released from carboxymethylcellulose hydrogel (H1 + oEV) or chitosan hydrogel (H2 + oEV) after 24, 48, or 72 h. Results are shown as the percentage of cell migration at 24 h from the scratch (t = 0). VEGF and EGF were used as positive controls. All histograms show the mean ± SEM from *n* = 4 experiments. In (**D**,**E**), the statistical significance was calculated by comparing all conditions vs. NT, and H1/2 vs. H1/2 + oEV. ns: not statistically significant, * *p* < 0.05, ** *p* < 0.01, *** *p* < 0.005, and **** *p* < 0.001.

Before in vivo testing on a wound healing model, we investigated the oEV formulation in order to understand the influence of hydrogel incorporation on oEV release and availability. oEVs were formulated with a carboxymethylcellulose and a chitosan hydrogel. By analyzing the release of oEVs (Figure 6A,B) we observed that oEVs were progressively released by both hydrogels from 30 min to 72 h, at a comparable level for the two hydrogels. Next, oEV uptake into target cells was monitored at 6, 24, 48, and 72 h comparing oEV alone and oEV released by carboxymethylcellulose hydrogel (Figure 6C). The uptake was significantly higher for oEV alone at 6 and 24 h, but it was similar or higher for oEV released from hydrogel at 48 h and significantly higher for oEV released from hydrogel at 72 h. This confirms that hydrogel can prolong the availability of oEVs over time.

The biological activity of oEVs released by hydrogels was also tested. oEVs released by both hydrogels at 24, 48, and 72 h showed pro-angiogenic activity by significantly increasing the formation of capillary-like structures compared to untreated cells and hydrogel alone in a tube formation in vitro assay (Figure 6D). Accordingly, oEVs released by both hydrogels at 24, 48, and 72 h showed a pro-regenerative effect by significantly promoting the migration of endothelial cells compared to untreated cells and hydrogel alone in a scratch test in vitro assay (Figure 6E).

### 3.6. In Vivo Test of Different oEV-Hydrogel Formulations on Skin Ulcers in Healthy and Diabetic Mice Models

**Figure 7:**
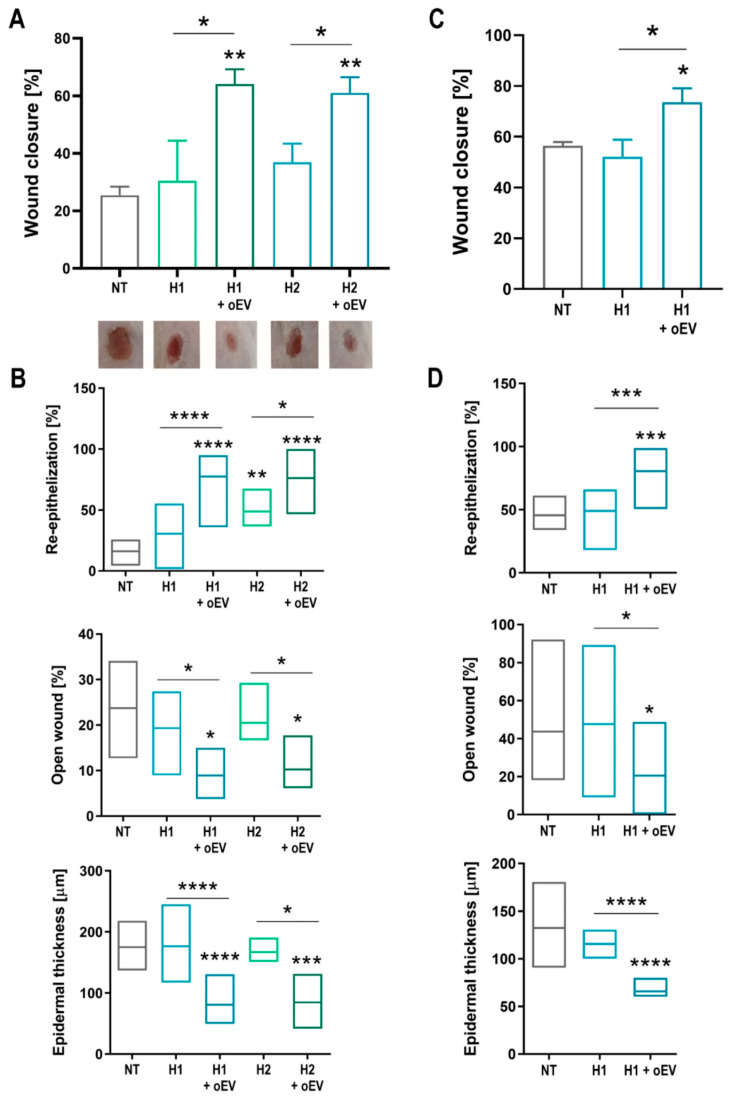
Test of different hydrogel formulations in vivo. (**A**) Percentage of wound closure of ulcers on healthy mice untreated (NT) or treated with hydrogel alone (H1/2) or hydrogel and 10^9^ oEVs (H1/2 + oEV). The insets show representative wounds on day 10 after wound onset. (**B**) Histological analysis of wounds from healthy mice showing the percentage of re-epithelization, the percentage of open wound, and the measure of epidermal thickness. Results of *n* = 3 mice per group. (**C**) Percentage of wound closure of ulcers on diabetic mice at day 14 after wound onset. (**D**) Histological analysis of wounds from diabetic mice showing the percentage of re-epithelization, the percentage of open wound, and the measure of epidermal thickness. Results of *n* = 3 (NT) and *n* = 4 (H1 and H1 + oEV) mice per group. Abbreviations: H1, carboxymethylcellulose hydrogel; H2, chitosan hydrogel. The graphs show mean ± SEM (**A**,**C**) and min to max values with the line at mean (**B**,**D**). The statistical significance was calculated by comparing all conditions vs. NT and H1/2 + oEV vs. H1/2. * *p* < 0.05, ** *p* < 0.01, *** *p* < 0.005, and **** *p* < 0.001.

To evaluate the potential therapeutic activity of oEVs, a wound-healing process was monitored on a punch-induced ulcers in the first experimental group of healthy (BALB/c) mice. After the ulcer induction, mice were treated with 1 × 10^9^ oEVs in carboxymethylcellulose (H1) and chitosan (H2) hydrogels, in order to choose the best hydrogel for further experiments. Wound closure monitored after 7 days showed a significant healing effect of oEVs formulated with both hydrogels with respect to the hydrogel alone (Figure 7A). The histological analysis showed significant re-epithelization, reduction of open wound and epidermal thickness induced by oEVs formulated with both hydrogels with respect to the hydrogel alone (Figure 7B). No prominent differences were observed between the two hydrogels, however, carboxymethylcellulose hydrogel was selected for the next experiments, because it was slightly less efficient in promoting wound healing *per se* with respect to chitosan hydrogel, allowing a better evaluation of the oEV efficacy. A group of (NGS) mice was treated with streptozotocin to induce diabetes, then the punch-induced ulcers were created, and the wound healing process was monitored until day 14 (Figure 7C). oEVs showed a significant healing effect compared to the hydrogel alone. The histological analysis showed no improvement with hydrogel alone compared to untreated (NT) ulcers, while oEVs induced a significant re-epithelization, reduction of the open wound, and epidermal thickness (Figure 7D).

### 3.7. In Vivo Test of Increasing Doses of oEV-Hydrogel Formulations on Skin Ulcers of Healthy and Diabetic Mice Models

**Figure 8:**
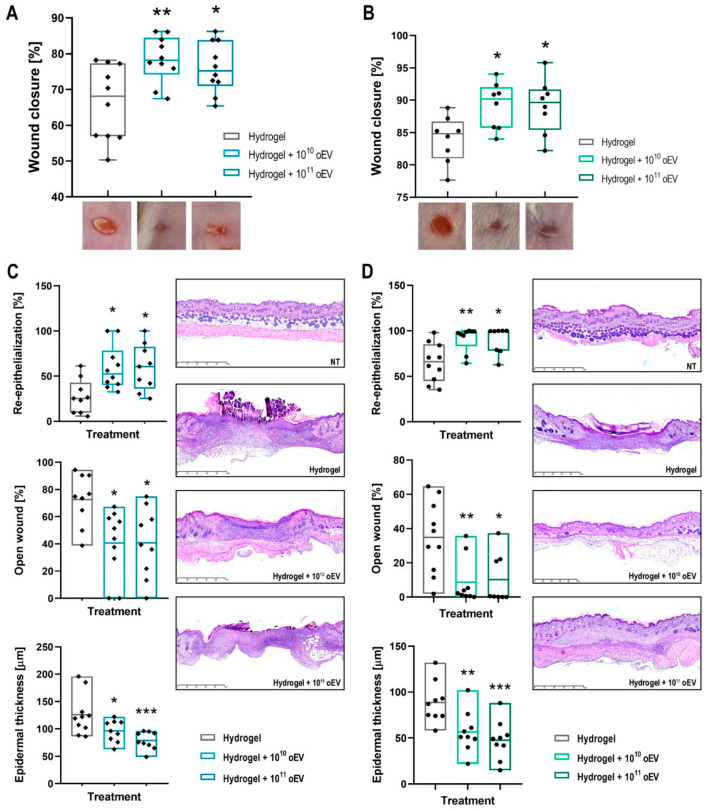
Test of increasing oEV doses in vivo. (**A**) Percentage of wound closure of ulcers on healthy mice treated with hydrogel alone or hydrogel and 10^10^ or 10^11^ oEVs. (**B**) Percentage of wound closure of ulcers on diabetic mice. The insets show one representative wound for each condition. (**C**) Histological analysis of wounds from healthy mice showing the percentage of re-epithelization, the percentage of open wound, and the measure of epidermal thickness. (**D**) Histological analysis of wounds from diabetic mice showing the percentage of re-epithelization, the percentage of open wound, and the measure of epidermal thickness. The insets show one representative H&E-stained section for each condition. Results of *n* = 10 mice per group on day 10 after wound onset. Hydrogel: carboxymethylcellulose hydrogel. The graphs show mean ± SEM (**A**,**B**) and min to max values with the line at mean (**C**,**D**). The statistical significance was calculated by comparing all conditions vs. hydrogel alone. * *p* < 0.05, ** *p* < 0.01, *** *p* < 0.005.

The in vivo wound healing was modelled on new experimental groups of wild-type (BALB/c) and diabetic (NGS) mice in order to test increasing doses (10^10^ and 10^11^ oEV) of oEVs embedded in carboxymethylcellulose hydrogel. Also at these doses, oEVs induced a significant wound closure on both healthy (Figure 8A) and diabetic mice (Figure 8B) but increasing the doses did not increase the healing effect. The histological analysis confirmed significant re-epithelization, and reduction of open wound and epidermal thickness induced by oEVs on healthy (Figure 8C) and diabetic mice (Figure 8D).

Moreover, no signs of toxicity were observed throughout the treatment as shown by unaltered body weight of healthy and diabetic mice (Figure S3C,D).

## 4. Discussion

Previous studies have shown that EVs derived from various cell sources, including stem cells [68] endothelial cells [69], fibroblasts, and human serum [70], possess pro-angiogenic and wound healing properties in vivo. However, studies on the effects of plant-derived extracellular vesicles showing the effects in in vitro and in vivo wound healing models have multiplied in later years, highlighting the ability of EVs from diverse plant sources to induce regenerative processes through promotion of cell proliferation, migration, vessel formation, reduce inflammation etc, either by acting directly onto damaged cells or tissues, or by activating endogenous effectors such as macrophages [71]. In addition, several advantages of PDNV mostly referring to their availability, scalability, and safety, make them appealing for therapeutic applications. Edible plants are an abundant natural source of EVs that can easily allow for high-yield EV extraction on a large scale when compared with EVs extracted from human stem cell conditioned medium. In the present study we report the intrinsic regenerative properties of EVs that are particularly abundant in the orange juice, from which they can be extracted through a low-cost and scalable process. Herein, we demonstrated that oEVs extracted from Citrus sinensis juice with TFF are morphologically and physio-chemically comparable to oEVs isolated by ultracentrifugation, the most common technique used to-date for EV isolation from different biological samples. TFF is a widely adopted, suitable, and highly effective method for both pharmacological grade and large-scale manufacturing of various biological products. Biophysical and biochemical properties remained unaltered in TFF-oEVs compared to UC-oEVs, with the only observable difference consisted in a statistically significant reduction in the protein content in the former. Since this was not associated with a reduction in the overall oEV recovery, we hypothesized that removed proteins are not associated with oEVs, but are usually co-precipitated during ultracentrifugation, while removed during the diafiltration step of TFF. Since no significant differences were observed in biological activities between UC-oEVs and TFF-oEVs we can infer that non-vesicular proteins were irrelevant for the observed bioactivities. Thus, we can suggest that TFF is suitable for the isolation of oEVs on a large scale, allowing a good recovery of oEVs with consistent characteristics and low protein contaminants.

In addition to the standard characterization parameters, we analyzed several oEV preparations by Raman spectroscopy observing very reproducible spectra between different batches. When the oEV Raman spectra were compared to those obtained on simple buffer solutions, new vibrational features were clearly observable. All the oEV samples showed four main peaks at 676, 710, 1416, and 1634 cm^−1^. Based on previous Raman evidence on citrus plants, the peak in the 1400–1500 region could correspond to CH_2_ bending mode of protein and lipids [72], or to scissoring and twisting vibrations of the CH_2_ groups typical of lipids [73]. Even the peaks in the 700 and 1600–1700 regions are compatible with lipids spectra previously observed [72,73]. Other findings suggest that peaks at 750 and 1600–1700 are associated with citrus flavonoids, such as hesperidin, and essential oils [74,75]. Interestingly, the presence of these molecules was confirmed by metabolomic analysis. Although further experiments should be performed to clearly identify the molecules that generate the observable peaks, Raman spectroscopy appears to be a suitable technique for a rapid and affordable characterization analysis of oEVs and tracing the reproducibility of production process.

Lipidomic analysis showed that the lipid composition of oEVs is very consistent in different oEV preparations. Looking at the lipid classes identified by the analysis, oEVs contain prevalently hexosylceramides, followed by phosphatidylethanolamines, phosphatidylcholines, phosphatidylinositol, and phosphatidylserines. Hexosylceramides serve as essential structural elements in mammalian cell membranes and lipid rafts. These compounds function as crucial intermediates in dihexosylceramide biosynthesis, which leads to the formation of more complex glycosphingolipids, including globosides and gangliosides. Hexosylceramides play vital roles in maintaining myelin structure, function, and long-term stability, while also supporting neuronal axonal development. Additionally, certain hexosylceramides perform the important function of preventing water loss through the epidermis. It also seems that membranes with high levels of hexosylceramides are more resistant to cryopreservation [76]. This might explain our observations that oEVs are more stable and resistant to cryopreservation and long-term storage (+4 °C/RT) than human-derived EVs. Their high stress resistance also allows for lyophilization and storage at room temperature without losing integrity or therapeutic potential [77]. These characteristics candidate oEV as potential drug delivery systems, on the top of leveraging their native biological properties. Phosphatidylethanolamines, phosphatidylcholines, phosphatidylinositol, and phosphatidylserines are the phospholipids more represented in cells, including human cells. The high presence of these lipids in EVs may explain, at least in part, the high biocompatibility of oEVs with human cells. Additionally, phosphatidylcholine and phosphatidylethanolamine were shown to have antioxidant, and anti-inflammatory effects, possibly supporting their therapeutic roles [71].

Metabolomic analysis showed the presence of several amino acids in oEVs, especially arginine, asparagine, and citrulline. Interestingly, arginine and citrulline were shown to play a favorable role in wound healing because they are involved in the arginase pathway, producing polyamines needed for cell proliferation and collagen synthesis. Moreover, arginine and glutamine activate the inducible nitric oxide synthetase (iNOS) pathway, which produces nitric oxide (NO) that regulates cell proliferation, collagen formation, and wound contraction [78,79]. A recent meta-analysis has demonstrated that arginine and glutamine dietary supplementation can promote wound healing, or parameters related to healing, including nitrogen balance and patient mortality [79]. In addition, evidence suggests that asparagine is downstream of glutamine’s pathway and contributes to maintaining endothelial cell growth and homeostasis [80].

Among the other metabolites, as expected, oEVs contain known antioxidant molecules, such as citric and ascorbic acid. Moreover, they contain limonene, which was shown to have antioxidant activity by promoting the activities of anti-oxidant enzymes superoxide dismutase (SOD) and catalase (CAT) in diabetic rats [81], and to have anti-inflammatory effects in murine dermal inflammation. Along with tissue-repair and angiogenesis modulatory properties [82]. Limonene and naringin also showed a protective effect against H_2_O_2_-induced DNA damage [83]. The high antioxidant capacity is confirmed by the high levels of ORAC, measuring the oxygen radical absorbance capacity.

Interestingly oEVs were shown to contain rutin, hesperidin, and naringin, all flavonoids that are known to have therapeutic potential for diabetic wound healing [84]. Rutin was shown to ameliorate wound healing and diabetes outcomes after intraperitoneal injection in streptozotocin-induced diabetic mice [85]. Hydrogels containing rutin were shown to reduce the wound area and decrease lipid peroxidation and protein carbonylation while increasing catalase activity in rat models [86]. Naringin was shown to enhance the proliferation and tube-formation capacity of endothelial progenitor cells in vitro, probably by activating PI3K/Akt signaling pathway [87]. Evidence showed that naringin has a pro-angiogenic effect on human umbilical vein endothelial cells (HUVECs) [88], promotes keratinocyte migration [89] and promotes wound healing in vivo [90] and the up-regulation of VEGF and VEGF-R3 [91].

These data overall suggest that the metabolites contained in oEVs may have a synergic effect in promoting angiogenesis and wound healing. The lower metabolite levels observed in TFF-oEVs compared to UC-oEVs could be due to the presence of free metabolites that co-precipitate with UC-oEVs and that are cleared throughout the TFF process. However, the overall concentration profile is very similar for the two oEVs, confirming their similarity. In a similar way in which human stem secretome that contains EVs is often considered more desirable for therapeutic applications than ultra-pure EVs, as it includes a full complement of bioactive factors and a broader spectrum of regenerative properties [92], it is possible that for some therapeutic applications of oEVs, and PDNV in general, modulating the overall purity through methodological choices, will be beneficial. This warrants further studies in additional use cases.

The proteomic analysis of oEVs, in part validated by WB analysis, evidenced several well-represented proteins. Abundant oEVs protein class is that of transmembrane proteins with catalytic ATPase activity involved in proton (H+) transport, such as proton pump ATPase 10 or V-ATPase, also known as AHA5; Vacuolar proton pump VHA-A, and H(+)-exporting diphosphatase AVP1. They are probably involved in vacuole acidification in orange fruit [90,91]. Other transmembrane transporter proteins are Nodulin-like domain-containing protein (NodGS), and the metal transporter NRAMP3. NodGS was initially identified in legumes as genes expressed upon nodulation induced by Rhizobium bacteria, “nodulin” proteins have been found in several species (i.e., Arabidopsis, rice, maize). Recent research emphasizes the critical role of nodulin-like proteins in transporting nutrients, solutes, amino acids, and hormones-functions essential to plant development [93]. These diverse integral membrane proteins facilitate the movement of water and small solutes across cellular membranes [94]. In Arabidopsis, AtNRAMP3 is found in the vacuole membrane where it regulates Fe-starvation-dependent accumulation mechanisms for Zn, Mn, and Fe by controlling metal transport through the vacuolar membrane [95].

oEVs contain other transmembrane proteins involved in signal transduction, such as Leucine-rich protein kinase domain-containing protein (LRR-RK), also known as EFR. In In Arabidopsis, the EFR receptor identifies bacterial EF-Tu through its interaction with elf18, a Pathogen Associated Molecular Pattern (PAMP) molecule [96].

Another well-represented protein class is ubiquitin and ubiquitin-related proteins, such as ubiquitin-NEDD8-like protein/RUB2, polyubiquitin UBQ11, and ubiquitin fused to a ribosomal protein RPS27AA. Ubiquitin-NEDD8 has been identified as playing a regulatory role in various developmental processes, including embryogenesis [97].

We also found chaperon proteins, such as DNAJ heat shock N-terminal domain-containing protein (DNAJ), a subfamily of Hsp40 proteins, Chloroplast Hsp70-2 protein, and Protein disulfide isomerase (PDI5). Hsp40/Dnaj protein serves as a cofactor for Hsp70, regulating ATPase activity and enabling reversible attachment to partially denatured protein substrates. This process prevents these substrates from aggregating with themselves or other molecules [98]. Protein disulfide isomerase (PDI) functions as a thiol-disulfide oxidoreductase that catalyzes oxidation, reduction, and isomerization reactions during protein folding and unfolding processes. Additionally, certain PDIs exhibit chaperone capabilities that assist proteins in achieving proper folding [99].

oEVs were shown to contain structural proteins such as actin (ARP7), clathrin (CHC1), SNARE protein (SYP121), and tetraspanin (TET8). SNARE (soluble N-ethylmaleimide-sensitive factor attachment protein receptor) proteins are essential for vesicle fusion and the transportation of membrane and cargo proteins within cells [100,101,102]. In plants, these proteins support critical functions including cell growth, homeostasis, protection against pathogens, and developmental processes. SYP121 and related SNARE proteins are broadly distributed throughout vegetative plant tissue and facilitate much of the secretory activity. Tetraspanin proteins organize membrane nanodomains involved in cell adhesion and migration. Their distinctive conical structure, combined with their capacity to interact with transmembrane receptors and connect with cytoskeletal and signaling frameworks, enables them to control endosomal network dynamics, extracellular vesicle (EV) formation, and cargo selection. The identification of TET8 tetraspanin indicates similarities between plant EV biogenesis and mammalian EV formation, particularly regarding the involvement of ESCRT genes.

Finally, we also observed high expression of Tobamovirus multiplication 2A (TOM2A) protein, a four-pass transmembrane protein that is required for the efficient multiplication of tobamovirus in tobacco and Arabidopsis [103]. This protein has no established role in EVs and their regenerative properties, it is known as a plant protein implicated in virus susceptibility.

We consider the results of this proteomic analysis as preliminary, due to the to-date lack of information on citrus protein databases and the lack of antibodies directed against citrus proteins for proper orthogonal data validation. Still, it shed new light on the oEV protein content and detected several proteins that are well expressed in all tested oEV preparations that could be used for oEV identification and characterization. No differences were observed between EVs isolated with UC and TFF, suggesting that the two isolation techniques do not select different subpopulations of EVs.

The RNA sequencing showed a high presence of rRNA in oEVs and GO Enrichment analysis on the first 100 most expressed genes showed a correlation to protein translation and ribosomal functions. These results suggest the presence of cytoplasmic RNA in oEVs. Prior studies in EVs isolated from plant tissues and cultures, reported the vesicular enrichment of mostly small RNA species, that were distinct with respect to RNA content of plant cells [104]. This, together with the detection of vacuolar proteins such as VHA-A, may support our hypothesis that part of the oEVs is generated with the mechanical disruption of cells and vacuoles when oranges are squeezed to produce the juice, representing the isolation artefacts, rather than naturally occurring EVs. To avoid the nomenclature issues raised by destructive purification procedures and no clear method to distinguish the true native plant EVs released in apoplastic fluid, from the artifacts or intracellular vesicles in the final preparation, we decided to adhere to ISEV suggestions [105], and adopt the term “plant derived nanovesicles” PDNV throughout this article. The PDNV, or oEVs, both indicate the comprehensive vesicle population recovered from the orange juice.

Both mRNA and miRNA analysis showed that the expression profiles of UC- and TFF-oEVs were similar, confirming that the TFF process does not affect oEV composition. Interestingly, among the most expressed mRNAs, we observed actin-7 (LOC102577980) and the heat shock proteins HSP90 and dnaJ protein homolog (LOC102608938), as well as the proteomic analysis has shown ARP7 (actin-7), HSP70-2, and DNAJ. Other mRNAs code for proteins involved in stress response, such as the probable inactive poly [ADP-ribose] polymerase SRO5 (LOC102619500), which is expressed in response to salt stress [106], DHN, and LEA5, which are involved in response to cold and drought stress [107,108], and thioredoxin H-type (LOC102624646), which is up-regulated in response to abiotic stresses and promotes abiotic stress tolerance by regulating redox homeostasis [109].

The analysis also revealed the presence in oEVs of a subset of novel miRNAs, some of which do not align with the assembly, while others do align but are not annotated. Some miRNAs (among both previously annotated and novel ones) showed an interesting similarity with human miRNAs. E.g., novel88, that is the second most expressed miRNA in oEVs, might mimic the hsa-miR-4749-5p, which was found in human EVs [110]. Of note, novel88 aligns with the genome of Medicago arabica, Oxyria digyna, and Lotus japonicus, thus we can hypothesize that the genomic locus of novel88 is not included in the available genome assembly of C sinensis. Further investigation is required to evaluate this hypothesis.

At the moment, no studies or function predictions are available for csi miRNAs, thus we carried out a prediction analysis. Interestingly, the predicted function of the miRNA novel73 is homophilic cell adhesion via plasma membrane adhesion molecules (GO:0007156); the predicted function of csi-miR390a-5p are focal adhesion (GO:0005925), the cell-substrate junction (GO:0030055), caveola (GO:0005901), leading edge membrane (GO:0031256); finally, the predicted function of novel90 are actin filament bundle (GO:0032432) and lamellipodium (GO:0030027).

Overall, the insight in molecular composition of oEVs revealed plenty of molecules that are likely to mediate a wide range of biological activities, similarly and in line with recently reported beneficial therapeutic effects exerted by PDNV from different plant sources [24,25,26,27,28,32,33,34,35,36,37]. The evidence got accumulated over last few years supporting the anti-tumor, anti-inflammatory, and antioxidant effects of EVs from edible plants. No adverse effects have been reported in any of these studies, while many of them report the impact of edible plants EVs on maintaining physiological intestinal homeostasis [111]. Our present study showcases oEVs as appealing therapeutic candidates for topical application to promote in vivo wound healing.

The in vitro studies have shown that oEVs stimulate human keratinocyte and endothelial cell motility favoring the closure of a wound induced in cell monolayers. oEVs promoted the organization of endothelial cells in vessel-like structures with an efficacy comparable to VEGF. Similarly, previous studies have shown that grapefruit-derived EVs are able to activate cell migration and vessel-like formation on HUVECs in vitro [112]. Vessel-like formation has been also reported for wheat-, lemon-, aloe- and cactus-derived EVs [34,113,114]. Of note, TFF-oEVs showed a pro-angiogenic effect comparable to UC-oEVs, suggesting that the biological effect, as well as the oEV physiochemical properties and the molecular components, are preserved with this isolation technique. In addition, oEVs were shown to promote the proliferation of endothelial cells cultured in hypoxic conditions, mimicking the oxygen reduction that occurs at the wound site.

Given the consistent presence of metabolites with potential antioxidant activity, such as ascorbic acid, citric acid, polyphenols, rutin, and naringin in oEVs, and the high ORAC level, we decided to test the antioxidant activity in various in vitro models. The experiments confirmed that oEVs retain a strong antioxidant activity that protects endothelial cells from oxidative stress induced by stimulation with hyperglycemia, alternated hyper and hypoglycemic insult, and an inflammatory cytokine. oEVs were able to reduce protein carbonylation induced by hyperglycemia. In endothelial cells treated with pro-inflammatory cytokines, oEVs dose-dependently reduced total and mitochondrial reactive oxygen species (ROS) levels, as well as lipid peroxidation and protein carbonylation. On the other hand, we observed an increase in reduced glutathione (GSH) levels and a reduction in oxidized glutathione (GSSG). In the presence of oEVs, the activity of the antioxidant enzymes G6PD, SOD1, SOD2, GR, GPX, and TrxR were significantly reduced. This may suggest that oEVs act by preventing or rapidly counteracting the formation of ROS without the consumption of GSH and the activation of the antioxidant enzymes, or by quickly restoring cell homeostasis. This could be due, for instance, to the mechanism of action of ascorbic acid, which prevents oxidation by scavenging free radicals [115] or by the synergistic effects of various antioxidant molecules. Several other in vitro studies support the antioxidant and anti-inflammatory properties of plant-derived EVs. A recent study [116] demonstrated that *Aloe saponaria*-derived EVs significantly reduce the expression of pro-inflammatory genes such as interleukin-6 and interleukin-1β by macrophages stimulated with lipopolysaccharide. Furthermore, *Aloe vera-derived* EVs exhibited antioxidant properties by promoting in vitro migration of keratinocytes and fibroblasts and by upregulating the expression of Nrf2, HO-1, CAT, and SOD genes in HaCaT keratinocyte cells treated with hydrogen peroxide [117]. The beneficial action of plant-derived EVs is not restricted to the skin, as they were shown to inhibit ROS generation in alcohol-induced liver injury [118] and to inhibit inflammatory and oxidative injury in various models of gastrointestinal injury [119,120,121,122]. We already mentioned that edible PDNVs can increase the production of genes for anti-inflammatory cytokines and antioxidant molecules that keep the intestines balanced [33].

With a view to applying oEVs on skin wounds in vivo, oEVs were formulated with different hydrogels for testing the gain in their sustained availability and functionality. In vitro studies demonstrated the prolongated release of oEVs from different hydrogels supporting the suitability of hereby studied preparations for topic application. oEVs were gradually released for up to 72 h, and were taken up by recipient cells, while retaining a pro-angiogenic and pro-migratory ability.

Next, oEVs were tested in vivo on skin wounds generated on the backs of healthy and diabetic mice. Two types of hydrogels were compared, carboxymethylcellulose and chitosan hydrogels, but no significant difference was observed between the two hydrogels. Instead, in both cases, oEVs were shown to accelerate wound closure, re-epithelization, reduction of open wound, and epidermal thickness at the histological level compared to the hydrogel alone. A similar result was observed in diabetic mice. Diabetes mellitus is a primary contributor to impaired angiogenesis resulting in diminished wound healing capacity. In diabetic patients, the impaired innate healing process not only depends on compromised vascularization, but also on multiple cell dysfunctions, including unregulated oxidative stress and sustained inflammation [123]. For this reason, we hypothesize that the antioxidant and pro-angiogenic activity of oEVs are particularly helpful in diabetic conditions. Moreover, we tested two increasing doses of oEVs in both healthy and diabetic mice. Both doses (10^10^ and 10^11^ oEVs/wound) induced significant wound closure and amelioration of the histological parameters, suggesting that the maximum effect is reached already with the dose of 10^10^ oEV/wound, while the higher doses did not show additional beneficial effects. The molecular evaluation of oEVs revealed multiple components with anti-inflammatory, anti-oxidative, and regenerative properties. These findings align with in vitro studies demonstrating that oEVs can enhance blood vessel formation, reduce oxidative damage, and support epithelial tissue renewal. Given this complexity, identifying the most significant biological mechanisms driving in vivo skin wound healing remains challenging. Consequently, our in vivo observations are primarily descriptive, as numerous factors may contribute to the healing process. It is reasonable to conclude that various elements work together to produce the ultimate biological effect.

Additionally, no adverse events or signs of cytotoxicity were shown in treated mice, suggesting that oEVs are safe and biocompatible. This is in line with our previous observation that mice treated with repeated doses of oEVs did not develop antibodies directed against oEVs (unpublished data). Therefore, while further ad hoc-designed studies should be performed to assess oEV safety, we can readily speculate that being a normal part of the human diet, they have an optimal safety profile, constituting an important advantage for their further therapeutic development and scaleup from a regulatory perspective.

## 5. Conclusions

In conclusion, oEVs are abundant extractive product of orange juice suitable for low-cost scalable production. Herein, we compared the purification of oEV isolated through ultracentrifugation and TFF showing no significant structural, physio-chemical, compositional, or functional differences. The acknowledged efficacy, adoption, and automatization feasibility of TFF purification supports a scalable and good practice compliant purification of oEVs for therapeutic and in-human applications.

The molecular characterization of oEVs shed new light on the previously unknown molecular content of oEVs, showing, among others, the presence of hexosylceramides, which may confer resistance to the EV membrane, and several antioxidants and anti-inflammatory molecules, especially flavonoids, which may be at the basis of the observed biological effect.

oEVs showed pro-regenerative activity on endothelial cells and keratinocytes in vitro, including pro-migratory, pro-angiogenic, pro-proliferative, and antioxidant effects. In vitro studies also demonstrated the prolongated release of oEVs from different hydrogel-based formulations, supporting the possibility of various preparations suitable for topic application. Moreover, oEVs accelerated the closure of skin wounds in both normal and diabetic mice, demonstrating the feasibility for topic application, enhanced by hydrogel usage. In addition, the pro-angiogenic activity observed in vitro could support the new vessel formation that is critical for wound healing, especially in diabetic conditions where angiogenesis is compromised.

Last but not the least, being a natural product, oEVs are inherently biocompatible and have not shown any cytotoxic effects, suggesting that they could present a useful tool for healing chronic wounds that require prolonged applications.

## 6. Patents

Some experiments reported in this manuscript are also reported in the Patent Application No. PCT/EP2021/074660 entitled “Pharmaceutical composition in the form of a hydrogel comprising orange-derived extracellular vesicles”.

## Figures and Tables

**Table 1:** Details of isolated different oEV batches.

Sample Name	Starting Volume	Final Volume	Concentration	Yield ^1^	Size [NTA]		Zeta Potential		pH		Protein Amount/oEV	RNA per 10^9^ EVs ^2^
	[ml]	[ml]	[EV/mL]	[EV/mL]	Mean [nm]	SD [nm]	Mean [mV]	SD [mV]	Mean	SD	[μg/10^9^ oEV]	[ng]
UC-oEV-1	1000	8.25	2.1 × 10^12^	2.2 × 10^10^	65.5	29.2	−15.13	0.91	3.38	0.00	1.024	3.06
TFF-oEV-1	5000	230	8.9 × 10^11^	4.7 × 10^10^	71.8	33.3	−18.55	1.26	3.23	0.01	0.684	2.25
UC-oEV-2	5000	32	2.9 × 10^12^	1.9 × 10^10^	64.8	31.3	−18.6	0.75	3.45	0.01	1.214	9.65
TFF-oEV-2	5050	207	1 × 10^12^	4.1 × 10^10^	80.2	36.5	−22.22	0.53	3.26	0.01	0.251	10.96
UC-oEV-3	2500	18.35	4.9 × 10^12^	3.6 × 10^10^	82.4	36.5	−23.29	0.93	3.45	0.01	0.647	4.41
TFF-oEV-3	5000	187	1.7 × 10^12^	6.4 × 10^10^	81.8	34.6	−23.32	0.51	3.36	0.01	0.227	2.26
UC-oEV-4	1000	6.1	2.4 × 10^12^	1.5 × 10^10^	82.6	34.7	−26.09	0.66	3.35	0.01	1.478	2.53
TFF-oEV-4	5000	162	6.7 × 10^11^	2.2 × 10^10^	86.8	36.3	−23.42	0.82	3.35	0.01	0.775	2.57
UC-oEV-5	1000	6.15	1.7 × 10^12^	1.0 × 10^10^	76.3	32	−26.09	0.66	3.35	0.01	1.524	2.53
TFF-oEV-5	5598	239.5	4.5 × 10^11^	1.9 × 10^10^	61.8	26.9	−28.66	1.17	3.28	0.02	1.243	3.98

^1^ Yield = total number of EV/starting volume of juice. ^2^ RNA per 10^9^ EVs = (RNA concentration × the RNA volume/starting EV number) × 10^9^ EVs.

**Table 2:** Full names and functions of the proteins detected in UC- and TFF-oEVs by Western Blot analysis. Information was retrieved in UniProt database [64].

Short Name	Full Name	Function
AHA5	ATPase 5 plasma membrane	Plasma membrane ATPase with catalytic activity, proton export across the plasma membrane
AVP1	Pyrophosphate-energized vacuolar membrane proton pump 1	H(+)-exporting diphosphatase, translocase, pyrophosphate hydrolysis-driven proton transmembrane transporter activity
DNAJ	Anti-DNAJ Heat Shock N terminal Domain (AT1G65280)	Heat Shock protein involved in stress response, similar to HSP70
EFR	LRR Receptor-Like Serine/Threonine-Protein Kinase EFR	Protein kinase domain-containing protein, ATP binding, protein kinase activity, protein phosphorylation
NEDD8-2	Ubiquitin-NEDD8-like protein RUB2	mRNA binding, ubiquitin protein ligase binding, protein ubiquitination
NodGS	Nodulin-like domain containing protein	Participates in the transportation of essential elements including nutrients, solutes, amino acids, and hormones, which are critical for key developmental processes in plants. Regulates plant–microbe interactions and host colonization.
SYP121	Syntaxin-121	Also referred to as Syntaxin related protein1/penetration1 (PEN1), this protein belongs to the SNARE family. These proteins facilitate vesicle fusion and play an essential role in the transportation of membrane and cargo proteins throughout the cell.
TOM2A	Tobamovirus Multiplication Protein 2A	Critical for the accumulation of Tobamovirus in Tobacco plants. TOM2A might play relevant roles in development or in plant responses to stresses, but no studies are available.
VHA-A	Vacuolar proton pump (H+)-ATPase (V-ATPase)- Subunit A	V-ATPase is a complex enzyme consisting of multiple subunits, with a peripheral V1 complex that breaks down ATP and an integral V0 membrane complex that moves protons. This enzyme plays a crucial role in acidifying and maintaining the pH levels within intracellular compartments.
ARP7	Actin-Related protein 7	Cytoskeleton structural protein
CHC1	Clathrin Heavy Chain 1	Structural protein that mediates the formation of lipid vesicles from the plasma membrane of cells
Hsp70-2	Chloroplast Hsp70-2	Heat Shock protein involved in stress response
TET8	Tetraspanin-8	Tetraspanin protein’s structure membrane nanodomains associated with cellular adhesion and motility.
PDI5	Protein disulfide isomerase-like 1-1	Protein Disulfide Isomerase (PDI) functions as a thiol-disulfide oxidoreductase enzyme that catalyzes the oxidation, reduction, and isomerization of target proteins. These processes are essential during protein folding and unfolding.
ABCC10 ^1^	ABC transporter C family member 10	ABC-type xenobiotic transporter, Catalytic Activity: ATP + H_2_O + xenobiotic = ADP + phosphate + xenobiotic.
NFD4 ^1^	MFS domain-containing protein, or MSSP1	The Major Facilitator Superfamily (MFS) is the largest group of secondary active membrane transporters. MSSP2 of Arabidopsis ia s sugar proton-coupled antiporter which contributes to vacuolar sugar import (e.g., monosaccharides including glucose, sucrose and fructose), particularly during stress responses (e.g., in response to cold).
NRAMP3 ^1^	Natural resistance-associated macrophage protein 3	Vacuolar transmembrane protein, metal transporter involved in intracellular metal homeostasis
FBA6 ^1^	Fructose 1,6-biphosphate aldolase	It is an enzyme involved in cytoplasmic gluconeogenesis and glycolysis, but also in plastids Calvin cycle
FBX6 ^1^	F-box domain-containing protein	The F-box functions as a motif involved in ubiquitin-dependent protein breakdown during cell cycle regulation and signal transduction processes. F-box proteins (FBPs) serve as the substrate-recruiting components of Skp1-cullin1-FBP (SCF)-type E3 ubiquitin ligases. These proteins play a crucial role in determining which specific proteins undergo ubiquitination.
UBQ11 ^1^	polyubiquitin 11-like isoform X1, or RPL40A	Ubiquitin is typically produced as a polyubiquitin precursor containing sequential head-to-tail repeats. In some cases, ubiquitin extension protein is produced with a single ubiquitin molecule attached to either a ribosomal protein (L40 or S27A) or a ubiquitin-related protein (RUB1 or RUB2). After translation occurs, the extension protein is separated from the ubiquitin.
RPS27AA ^1^	ubiquitin-40S ribosomal protein S27a-like	One molecule of ubiquitin linked to ribosomal protein S27A.

^1^ Protein identified but not tested by WB.

**Table 3:** miRNA function and target prediction.

miRNA	ID	Description	GeneRatio	BgRatio	p.adjust
novel73	GO:0007156	homophilic cell adhesion via plasma membrane adhesion molecules	18/90	172/21,081	3.16 × 10^−17^
csi-miR482e-3p	GO:0016936	galactoside binding	3/91	13/20,616	6.98 × 10^−3^
csi-miR482d-5p	GO:0048820	hair follicle maturation	3/92	16/21,081	8.42 × 10^−3^
novel85	GO:0014704	intercalated disc	4/91	51/21,872	1.31 × 10^−2^
csi-miR482e-3p	GO:0035739	CD4-positive, alpha-beta T cell proliferation	3/89	16/21,081	1.61 × 10^−2^
csi-miR390a-5p	GO:0031252	cell leading edge	9/96	449/21,872	2.50 × 10^−2^
csi-miR390a-5p	GO:0005925	focal adhesion	9/96	475/21,872	2.50 × 10^−2^
csi-miR390a-5p	GO:0030055	cell-substrate junction	9/96	483/21,872	2.50 × 10^−2^
csi-miR390a-5p	GO:0005901	caveola	4/96	92/21,872	4.23 × 10^−2^
csi-miR390a-5p	GO:0031256	leading edge membrane	5/96	187/21,872	4.23 × 10^−2^
novel90	GO:0032432	actin filament bundle	4/97	79/21,872	4.60 × 10^−2^
novel90	GO:0030027	lamellipodium	6/97	220/21,872	4.60 × 10^−2^
novel88	GO:0032526	response to retinoic acid	5/92	126/21,081	4.71 × 10^−2^

## Data Availability

The data are available in the manuscript, in the Appendix A, in the Supplementary Materials, in Zenodo https://doi.org/10.5281/zenodo.18233502 and in the Data Book held by EV Biosolution S.p.A.

## Supplementary Materials

The following supporting information can be downloaded at: https://www.mdpi.com/article/10.3390/cells15030244/s1, Figure S1: Analysis of long RNA identified in oEVs by RNA sequencing. (A) Read distribution on C. sinensis genome; Figure S2: Analysis of miRNA identified in oEVs by RNA sequencing; Figure S3: Mice weight and glycemia during diabetic induction and mice weight during treatment; Table S1: List of the antibodies used for western blots; Table S2: Geneid; Table S3: miRNA.

## Appendix A

**Table A1:** Results from proteomic analysis performed with 2-DE gel, MS, and Mascot analysis.

Number	Protein ID	Protein Name
1	TVU00056.1	H(+)-exporting diphosphatase
2	GFZ00503.1	Vacuolar proton pump subunit B
3	EOY27420.1	P-type H(+)-exporting transporter
4	GAY34894.1	Cation_ATPase_N domain-containing protein
5	TXG68154.1	P-type H(+)-exporting transporter
6	ESR35870.1	Nodulin-like domain-containing protein
7	BAK02751.1	Predicted protein
8	EOA18061.1	Uncharacterized protein
9	QOU08746.1	Ubiquitin-like protein RUB2
10	KAE9454244.1	F-box domain-containing protein
11	RLN34543.1	Ubiquitin-like

**Table A2:** Results from proteomic analysis performed with MS and Mascot analysis.

LC-MS/MS Identification	Protein ID	MW (Da)	Isoelectric Point (pI)
VHA1, partial [Vicia faba]	AAA81349.1	33,627	6.04
vacuolar H+-PPase protein [Suaeda corniculata]	ADQ00196.1	79,963	5.04
MRP-like ABC transporter, partial [Citrus reshni]	AGR67380.1	13,503	8.07
predicted protein [Hordeum vulgare subsp. vulgare]	BAK02751.1	17,752	9.8
predicted protein [Hordeum vulgare subsp. vulgare]	BAK08287.1	73,785	7.29
hypothetical protein VIGAN_08249300 [Vigna angularis var. angularis]	BAT95718.1	168,405	8.29
unnamed protein product [Arabidopsis thaliana]	CAD5325615.1	8136	5.71
Nodulin-like domain-containing protein [Citrus clementina]	ESR35870.1	43,206	4.85
hypothetical protein CICLE_v10025394mg [Citrus clementina]	ESR39256.1	49,927	5.16
Fructose-bisphosphate aldolase [Citrus clementina]	ESR43529.1	36,255	6.96
DNAJ heat shock N-terminal domain-containing protein [Actinidia rufa]	GFZ00491.1	42,940	9.29
ABC transporter C family member 10 [Morella rubra]	KAB1204962.1	164,390	8.1
putative xenobiotic-transporting ATPase [Lupinus albus]	KAE9598246.1	170,093	6.9
hypothetical protein CISIN_1g033708mg [Citrus sinensis]	KDO40488.1	12,930	5.8
hypothetical protein SOVF_071020 [Spinacia oleracea]	KNA18409.1	147,546	6.77
hypothetical protein DCAR_001907 [Daucus carota subsp. sativus]	KZN09251.1	105,660	6.25
Cation-transporting P-type ATPase [Corchorus olitorius]	OMO52600.1	105,646	6.35
hypothetical protein GOBAR_AA19551 [Gossypium barbadense]	PPS01112.1	103,419	6.17
ATPase [Arachis hypogaea]	QHO45754.1	104,617	5.74
ubiquitin-like protein RUB2 [Larix gmelinii var. olgensis]	QOU08746.1	17,182	5.97
ubiquitin-like [Panicum miliaceum]	RLN34543.1	16,286	9.89
hypothetical protein BHE74_00038226 [Ensete ventricosum]	RWW55155.1	39,706	8.99
hypothetical protein EJD97_011445 [Solanum chilense]	TMX04147.1	79,190	5.06
hypothetical protein EJB05_54549 [Eragrostis curvula]	TVU00056.1	80,025	5.32
hypothetical protein ES332_A13G137200v1 [Gossypium tomentosum]	TYH91778.1	67,978	5.74
hypothetical protein ES332_A10G056900v1, Plasma membrane ATPase [Gossypium tomentosum]	TYI04985.1	104,949	5.51
unnamed protein product [Triticum turgidum subsp. durum]	VAH00474.1	57,742	7.04
unnamed protein product [Brassica rapa]	VDD00160.1	68,654	5.16
ATPase 10, plasma membrane-type isoform X1 [Citrus clementina]	XP_006426822.2	104,898	5.79
ATPase 10, plasma membrane-type-like isoform X1 [Citrus sinensis]	XP_006465724.1	106,557	5.85
V-type proton ATPase subunit B3 [Brassica napus]	XP_013729997.1	54,121	5.08
ATPase 10, plasma membrane-type-like [Ziziphus jujuba]	XP_015867387.1	105,234	5.72
ubiquitin-40S ribosomal protein S27a-like [Ziziphus jujuba]	XP_015879285.1	17,788	9.42
V-type proton ATPase subunit B2-like [Coffea arabica]	XP_027088298.1	54,329	5
polyubiquitin 11-like isoform X1 [Triticum dicoccoides]	XP_037420296.1	30,985	9.03

**Table A3:** Results of novel C. sinensis (csi) miRNA alignment to human (hsa) miRNA for identifying possible csi miRNA mimicking the has miRNA.

csi-miR	hsa-miR (Link to Entrex Gene Data)	Num Common ENSG	Jaccard	Fisher *p* Value	Score	Average Expression	miRNA Mature Class	Alignment	References
csi-miR164a-5p	hsa-KmiR-5703	76	0.61	1.61 × 10^−120^	20	27.4	csi-miR164a-5p; csi-miR164b-5p; csi-miR164c-5p; csi-miR164d-5p	uGGAGAAgcagggcacgugca .|||||||..|||.|.|| aGGAGAAgucgggaaggu---	[107]
csi-miR3954	hsa-miR-4520-3p	26	0.15	1.01 × 10^−21^	21	10.9	csi-miR3954	-uGGACAGagaaaucacgguca ||||||||.||..|.|.|..| uUGGACAgaaaacacgcaggaa	[108,109,110]
csi-miR166b-3p	hsa-miR-10399-3p	12	0.06	1.84 × 10^−6^	20	173.4	csi-miR166b-3p; csi-miR166d-3p; csi-miR166g-3p	-uCUCGGAccaggcuucauucc ||||||||.||...|...|| cUCUCGGacaagcuguagguc-	[111]
csi-miR398b-3p	hsa-miR-4273	4	0.02	0.29	20	34.6	csi-miR398b-3p	uGUGUUCucaggucgccccug ||||||||.|.|.|.|.. -gUGUUCUcugauggacag--	[112]
csi-miR167d-5p	hsa-miR-22-3p	3	0.02	0.49	19	43.3	csi-miR167d-5p; csi-miR167e-5p	uGAAGCUgccagcaugaucua---- |||||||||| .|||...| --aAGCUGCcag-uugaagaacugu	[113]
csi-miR396b-3p	hsa-miR-7159-5p	1	0.01	0.73	20	10.5	csi-miR396b-3p	gUUCAAUaaagcugugggaag- |||||.||.|.|||.|||.| -uUCAACAaggguguaggaugg	-
novel88	hsa-miR-4749-5p	1	0.01	0.73	21	927.9	novel88	-gCGGGGCgaagccagagga— ||||||..|.|||||.|.| uGCGGGGacaggccagggcauc	[114,115]
csi-miR159a-3p	hsa-miR-4464	0	0	0.17	19	1068.6	csi-miR159a-3p	----uUUGGAUugaagggagcucua |||||||.||.|..|.. aAGGUUUggauagaugcaaua----	[110]
novel122	hsa-miR-6831-5p				22	42.1	novel122	----uAGAGUGagaggauguuggugag |||||||.|||||.|..|.. uAGGUAGagugugaggaggagguc---	
novel3	hsa-miR-1182				19	75.2	novel3_4	aUAGAUGgucuuuguaaggaag---- ||.||||||.|.|.|||.| ---gAGGGUCuugggagggaugugac	[116,117]
novel81	hsa-miR-3660				21	256.2	novel81	gAGUCUGacaugugagca------ .||||||.|.||||| ---aCUGACAggagagcauuuuga	[118,119]
novel85	hsa-miR-4474-5p				17	10.1	novel85_87_94	cCGAUUAgucucuguaacgugagca ||||||||...|.|.....|| ----uUAGUCUcaugaucagacaca	[110,120,121]
