# Supplementary material for: Orange-Derived Extracellular Vesicles: Characterization and Therapeutic Applications in Normal and Diabetic Wound Healing in In Vivo Models
Source: Cells. 2026 Jan 27;15(3):244. doi: 10.3390/cells15030244 (PMC12896877; doi:10.3390/cells15030244)
Supplement: Supplementary file 1 [file cells-15-00244-s001.zip › cells-4071149 260114 Supplementary.pdf]

Supplementary information

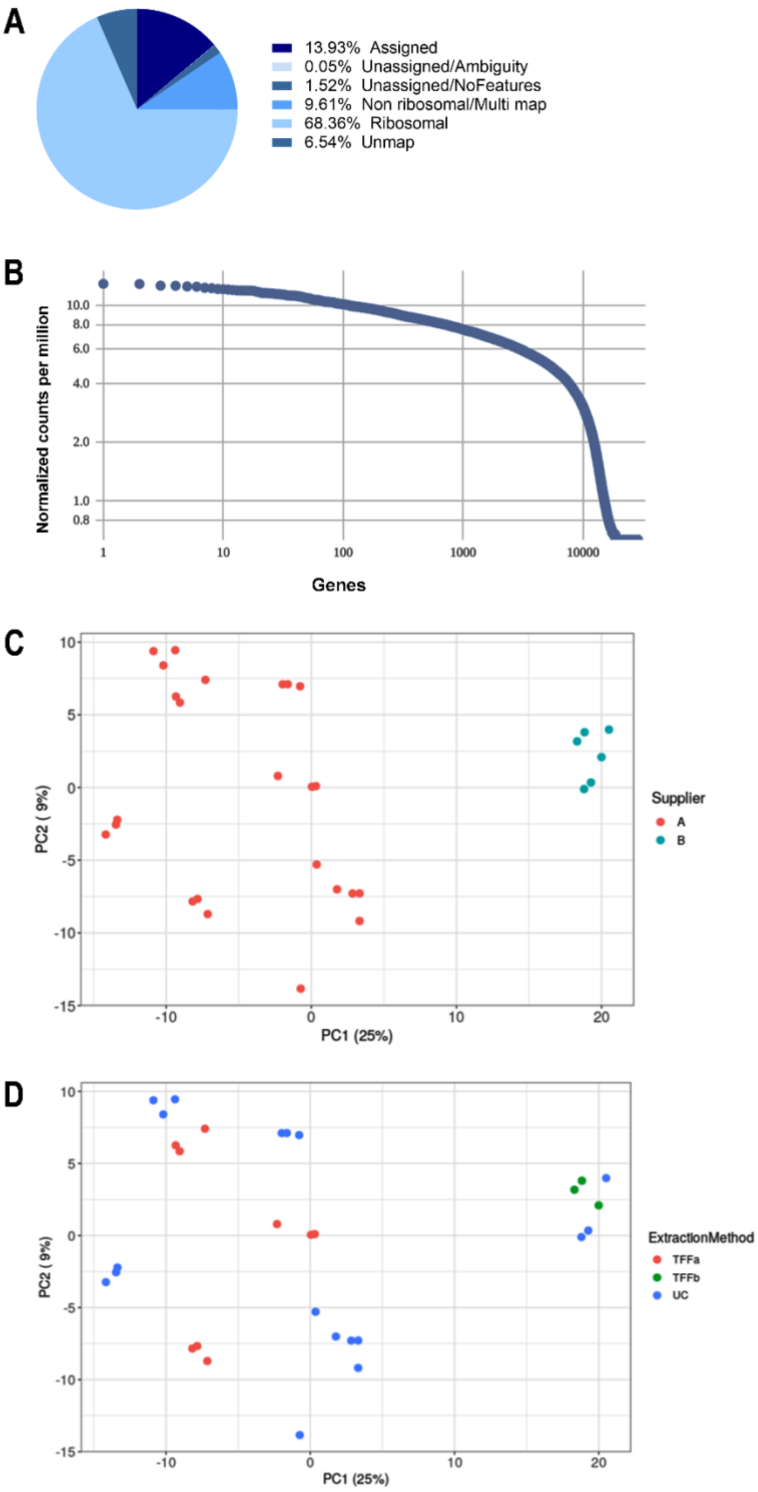

Figure S1. Analysis of long RNA identified in oEVs by RNA sequencing. (A) Read distribution on C. sinensis genome. The legend shows the percentage of reads matching with C. sinensis genes (Assigned), multiple loci (Non ribosomal/Multi map), Ribosomal genes, Unassigned, or not present in the genome (Unmap). (B) The graph shows the distribution of the number of reads for each gene (as normalized count per million of reads), mean of all samples. (C-D) Principal component analysis (PCA) analyzing the variability in oEVs obtained from different orange suppliers named A and B (C) and different isolation techniques (D), UC, TFFa (starting volume of 5 liters, supplier A), TFFb (starting volume of 50 liters, supplier B). Analysis of n=6 UC-oEV and n=4 TFF-oEV preparations run in triplicate.

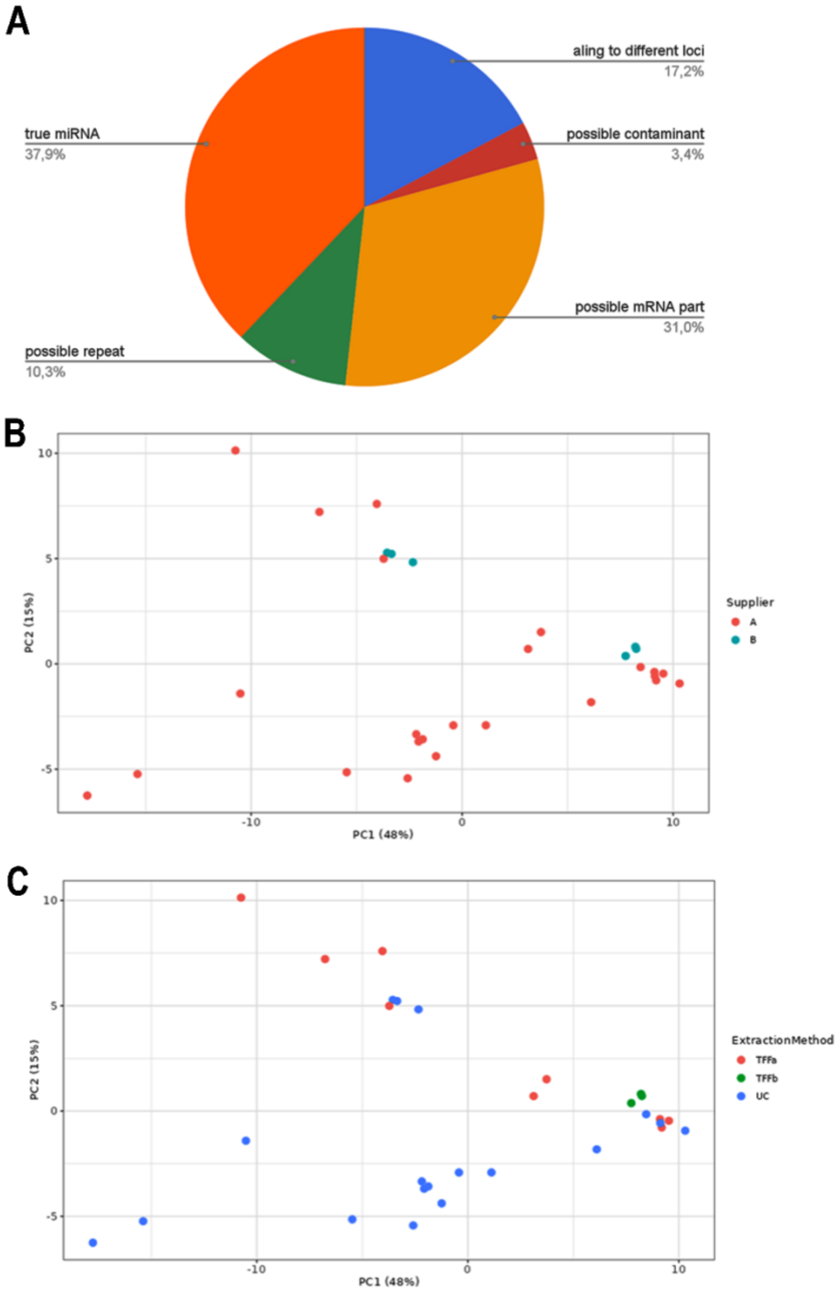

Figure S2. Analysis of miRNA identified in oEVs by RNA sequencing. (A) BLAST analysis of predicted novel pre-miRNA onto C. sinensis genome to identify probably false positive. (B-C) Principal component analysis (PCA) analyzing the variability in oEVs obtained from different orange suppliers named A and B (B) and different isolation techniques (C), UC, TFFa (starting volume of 5 liters, supplier A), TFFb (starting volume of 50 liters, supplier B). Analysis of n=6 UC-oEV and n=4 TFF-oEV preparations run in triplicate.

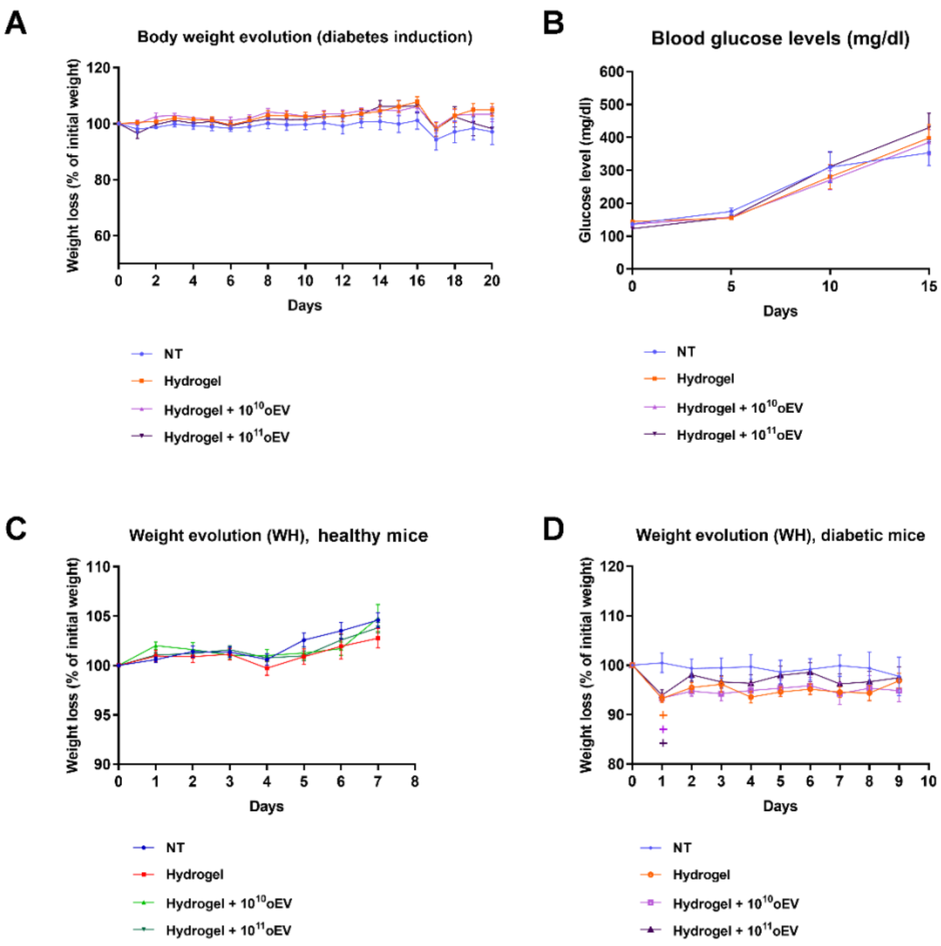

Figure S3. Mice weight and glycemia during diabetic induction and mice weight during treatment. (A) Mice's weight (expressed as a percentage of the initial weight) was monitored following the treatment with streptozotocin for diabetes induction. (B) Concentration of glucose (expressed as mg/dl) was measured in the mice's blood at days 0, 5, 10, and 15 following the treatment with streptozotocin to assess diabetes induction. (C) Mice's weight (expressed as a percentage of the initial weight) was monitored during the treatment with hydrogel and oEVs in healthy mice. (D) Mice's weight (expressed as a percentage of the initial weight) was monitored during the treatment with hydrogel and oEVs in diabetic mice. Data are expressed as mean ± SD.

Table S1. List of the antibodies used for western blots (shown in Figure 3A). All the antibodies were Polyclonal Antibodies produced in Rabbit against proteins of Arabidopsis thaliana, they were purchased from Phyto Ab (San Jose, CA, USA) and diluted 1:1000 in Every Blot Blocking Buffer (Bio-Rad, Hercules, CA, USA).

Short name	Antibody	Product number
AHA5	AHA5, Polyclonal Antibody	PHY2378A
AVP1	AVP1, Polyclonal Antibody	PHY0719A
DNAJ	AT1G65280 Anti-DNAJ Heat Shock N terminal Domain Antibody	PHY2619S
EFR	EFR / Anti-LRR Receptor-Like Serine/Threonine-Protein Kinase EFR Antibody	PHY1279S
NEDD8	NEDD8-2/ Anti-RELEATED TO UBIQUITIN 2 antibody	PHY0755A
NodGS	NodGS Antibody	PHY1045S
SYP121	SYP121 / Anti-Syntaxin-121 Antibody	PHY2912S
TOM2A	TOM2A / Anti-Tobamovirus Multiplication Protein 2A Antibody	PHY3962S
VHA-A	VHA-A, Polyclonal Antibody	PHY3322S
ARP7	ARP7 / Anti-actin-Related protein 7 antibody	PHY0974S
CHC1	CHC1 / Anti-Clathrin Heavy Chain 1 Antibody	PHY0227A
Hsp70-2	Hsp70-2 / Anti-chloroplast Hsp70-2 Antibody	PHY0788A
TET8	TET8 / Anti-Tetraspanin-8 antibody	PHY1490S
